# Are associations of adulthood overweight and obesity with all-cause mortality, cardiovascular disease, and obesity-related cancer modified by comparative body weight at age 10 years in the UK Biobank study?

**DOI:** 10.1038/s41366-025-01718-4

**Published:** 2025-01-23

**Authors:** William Johnson, Tom Norris, Natalie Pearson, Emily S. Petherick, James A. King, Scott A. Willis, Rebecca Hardy, Susan Paudel, Emma Haycraft, Jennifer L. Baker, Mark Hamer, David J. Stensel, Kate Tilling, Tom G. Richardson

**Affiliations:** 1 School of Sport, Exercise and Health Sciences, Loughborough University, Loughborough, UK; 2 National Institute for Health and Care Research (NIHR) Leicester Biomedical Research Centre, University Hospitals of Leicester National Health Service (NHS) Trust and the University of Leicester, Leicester, UK; 3 Institute of Sport Exercise and Health, Division of Surgery and Interventional Science, University College London, London, UK; 4 Institute for Physical Activity and Nutrition (IPAN), Deakin University, Burwood, VIC Australia; 5 Center for Clinical Research and Prevention, Copenhagen University Hospital-Bispebjerg and Frederiksberg, Copenhagen, Denmark; 6 National Institute for Health and Care Research (NIHR) University College London Hospitals Biomedical Research Centre, London, UK; 7 Faculty of Sport Sciences, Waseda University, Tokorozawa, Japan; 8 Department of Sports Science and Physical Education, The Chinese University of Hong Kong, Ma Liu Shui, Hong Kong, China; 9 MRC Integrative Epidemiology Unit (IEU), Population Health Sciences, Bristol Medical School, University of Bristol, Bristol, UK

**Keywords:** Epidemiology, Cardiovascular diseases, Cancer

## Abstract

**Objective:**

Adults living with overweight or obesity do not represent a single homogenous group in terms of mortality and disease risks. The aim of our study was to evaluate how the associations of adulthood overweight and obesity with mortality and incident disease are modified by (i.e., differ according to) self-reported childhood body weight categories.

**Methods:**

The sample comprised 191,181 men and 242,806 women aged 40–69 years (in 2006–2010) in the UK Biobank. The outcomes were all-cause mortality, incident cardiovascular disease (CVD), and incident obesity-related cancer. Cox proportional hazards regression models were used to estimate how the associations with the outcomes of adulthood weight status (normal weight, overweight, obesity) differed according to perceived body weight at age 10 years (about average, thinner, plumper). To triangulate results using an approach that better accounts for confounding, analyses were repeated using previously developed and validated polygenic risk scores (PRSs) for childhood body weight and adulthood BMI, categorised into three-tier variables using the same proportions as in the observational variables.

**Results:**

In both sexes, adulthood obesity was associated with higher hazards of all outcomes. However, the associations of obesity with all-cause mortality and incident CVD were stronger in adults who reported being thinner at 10 years. For example, obesity was associated with a 1.28 (1.21, 1.35) times higher hazard of all-cause mortality in men who reported being an average weight child, but among men who reported being a thinner child this estimate was 1.63 (1.53, 1.75). The ratio between these two estimates was 1.28 (1.17, 1.40). There was also some evidence that the associations of obesity with all-cause mortality and incident CVD were stronger in adults who reported being plumper at 10 years. In genetic analyses, however, there was no evidence that the association of obesity (according to the adult PRS) with mortality or incident CVD differed according to childhood body size (according to the child PRS). For incident obesity-related cancer, the evidence for effect modification was limited and inconsistent between the observational and genetic analyses.

**Conclusions:**

Greater risks for all-cause mortality and incident CVD in adults with obesity who perceive themselves to have been a thinner or plumper than average child may be due to confounding and/or recall bias.

## Introduction

In 2021, 26% of adults in England had obesity and a further 38% had overweight. This epidemic is a major public health concern, in part because adulthood obesity increases risk for numerous chronic diseases, including coronary heart disease (CHD), stroke, and different types of cancer [1–4]. Body mass index (BMI) and weight status are not, however, stable across the life-course, and disease risks related to adulthood overweight and obesity will vary according to factors such as age of onset and childhood weight status [5, 6].

Part of this literature has investigated how disease risks differ between four or more “trajectory” groups, defined based on the combination of childhood and adulthood weight status (e.g., overweight/obesity in childhood but not adulthood versus normal weight in childhood and adulthood) [7–11]. The seminal publication of Abraham et al. in 1971 showed that rates of some cardiovascular diseases (CVD) were highest among individuals who had overweight in adulthood but below average weight in childhood [12]. Several articles have replicated this type of analysis for CVD risk factors and outcomes [7–11, 13–17], and in 2020 this literature was summarised in a systematic review and meta-analysis [18]. In that paper, Sun et al concluded that “individuals who developed excess weight in adulthood or had excess weight in both periods had higher odds of developing CVD risk factors and incident outcomes in adulthood” [18]. This does not, however, necessarily mean that the association of adulthood obesity with CVD risk differs according to childhood weight status.

There is far less evidence for the 13 adulthood obesity-related cancers (breast, colon and rectum, corpus uteri, esophagus, gallbladder, gastric cardia, kidney, liver, meningioma, multiple myeloma, ovary, pancreas, and thyroid) identified by the International Agency for Research on Cancer [19]. A few papers have investigated the associations of overweight/obesity patterns between childhood and adulthood [20, 21], or the interactive associations of childhood body size and subsequent weight gain [22, 23], with risk of specific cancers, but none have considered obesity-related cancers collectively. There is also a dearth of knowledge for all-cause mortality. We are only aware of three studies that have considered childhood and adulthood BMI in relation to all-cause mortality [24–26], and none of these investigated how risk related to adulthood overweight or obesity is modified by childhood weight status.

Adjustment for adulthood lifestyle variables (potentially on the causal pathway from childhood weight status to adulthood weight status and disease risk) is common place in the literature, although this is generally not recommended because doing so can introduce collider stratification bias [27, 28]. Some degree of residual confounding is also highly likely regardless of what adjustments are made. Mendelian randomisation is an approach that can help deal with these challenges through the use of genetic variants as instrumental variables [29, 30]. Using data from the United Kingdom (UK) Biobank study, previous research has investigated the extent to which the associations of childhood body size with disease risk operates via adulthood body size [31]. In that paper, Richardson et al developed and used two polygenic risk scores (PRSs), one for adult BMI and one for childhood body weight, based on the question “‘When you were 10 years old, compared to average, would you describe yourself as: Thinner, About Average, Plumper”. The two PRSs were used within a multivariable Mendelian Randomization framework, with the key finding being that the positive associations of childhood body weight with odds of CHD and type two diabetes were largely mediated by adulthood BMI. This does not, however, mean that adults living with overweight or obesity have the same disease risks regardless of their childhood body weight. This is a separate research question that has not yet been addressed, for any outcome in any study, using PRSs.

Many studies in this literature interpret their findings through a prospective lens (i.e., can the disease risk associated with paediatric obesity be alleviated by becoming a normal weight adult). In her commentary on the Abraham et al study, when it was republished by the International Journal of Epidemiology in 2016 [32], Caroline Fall and colleagues described this as “forward-looking” [33]. Interpreting findings through a retrospective lens or “backward-looking” is, however, equally important, not least because most individuals will first present with obesity during adulthood [6, 33, 34]. Disease risk stratification might be possible by asking adults simple questions (e.g., were you a thinner or plumper than average child?) but evidence is lacking to support such a clinical recommendation because many studies do not explicitly test for effect modification of the association of adulthood obesity with the outcome(s) by childhood weight status [7–11]. Further, in clinical settings for adults with overweight or obesity, measured childhood BMI is unlikely to be available and self-reported weights (let alone heights) are unlikely to be accurate [35–37]. Adults are, however, more likely to be able to respond with reasonable certainty to a three-tier question about their body weight relative to their peers.

The aim of the present paper was to evaluate whether the associations of adulthood overweight and obesity with all-cause mortality, incident CVD, and incident obesity-related cancer differ according to comparative body weight at age 10 years. In addition to conventional analyses, including models adjusted for adulthood lifestyle variables (to allow comparison to the literature), we perform analyses less prone to confounding using PRSs.

## Methods

### Study

We used data from the UK Biobank, a prospective population-based cohort study that recruited over 500,000 adults aged 40–69 years between 2006 and 2010 [38]. The current study was conducted as part of the UK Biobank approved project 80843.

### Ethics and informed consent

Ethical approval was granted by the Northwest Multi-Centre Research Ethics Committee (ref. [11]:/NW/0382), and all participants provided written informed consent.

### Sample

Our sample comprised 191,181 men and 242,806 women with complete data (on the variables used in our analyses), together representing 86% of the full UK Biobank cohort.

### Outcomes

The three outcomes were all-cause mortality, incident CVD (ICD-10 codes I00–I99), and incident obesity-related cancer (ICD-10 codes C15 oesophagus, C16.0 gastric cardia, C18 colon, C19.9 and C20.9 rectum, C22 liver, C23 gallbladder, C25 pancreas, C54 and C55 corpus uteri, C56 ovary, C64 kidney, C70 meningioma, C73 thyroid, C90.0 multiple myeloma) obtained from administrative records (e.g., National Death Registries, Hospital Episode Statistics, and National Cancer Registries) [39]. Follow-up started at baseline and ended with an event or censoring: 19^th^ December 2022 for all-cause mortality, 1^st^ September 2023 for incident CVD, 15^th^ March 2022 for incident obesity-related cancer. Events (e.g., obesity-related cancers) occurring before baseline were not considered because adjustment for them might bias estimates due to conditioning on a mediator (i.e., a first cancer before baseline might be on the causal pathway between child body size and the outcome).

Breast cancer was not included in the obesity-related cancer outcome for two reasons. Firstly, higher childhood BMI may actually be protective for breast cancer [40]. Secondly, associations of adulthood BMI may differ in directions for pre-menopausal breast cancer and postmenopausal breast cancer [41]. Including breast cancer in the obesity-related cancer outcome would therefore obscure results.

### Exposure and effect modifier

Weight and height were measured at baseline. The exposure was weight status, defined as being normal weight (BMI 18.5–24.9 kg/m^2^), overweight (BMI 25.0–29.9 kg/m^2^), or living with obesity (BMI ≥ 30 kg/m^2^). The numbers were too low to consider different obesity classes (e.g., Class 3 = BMI ≥ 40 kg/m^2^). The 2,389 participants (0.5%) who were underweight (BMI < 18.5 kg/m^2^) were not included in our sample because combining them with the normal weight group would obfuscate the meaning of the referent group.

The effect modifier of interest was comparative body weight at age 10 years. At the baseline assessment, adults were asked “‘When you were 10 years old, compared to average, would you describe yourself as: Thinner, About Average, Plumper.”

In addition to measured adulthood BMI and self-reported comparative body weight at 10 years, we examined PRSs in a sub-sample with genetic data representing 97% of the full sample. The PRSs were developed from genome-wide association studies in the UK Biobank using the same variables studied the current paper (i.e., adulthood BMI and comparative body weight at 10 years). The PRSs have been validated in the Avon Longitudinal Study of Parents and Children, the Young Finns Study, and the Trøndelag Health study [31, 42, 43]. The adulthood BMI PRS comprised 579 variants and the childhood comparative body weight PRS comprised 312 variants. There is minimal overlap between the PRSs (i.e., 72 overlapping genetic loci) and previous work has demonstrated that the magnitude of estimates for many genetic variants in the PRSs differ with respect to early life and adult body size, thereby suggesting that they can be separated as two exposures [31]. Further description of how the PRSs were constructed has been published by Richardson et al. [31, 44]. In the main analyses, each PRS was categorised into a three-tier variable using the same proportions as in the observational variable (e.g., normal weight, overweight, obesity or about average, thinner, plumper).

### Covariates

Covariates were selected based on theoretical and epidemiological considerations. The core covariates included age at baseline assessment, ethnicity, and self-reported data on relative age voice break (males) or age at menarche (females) and comparative height at age 10 years. These variables are most likely potential confounders (of the exposure–outcome and effect modifier-outcome associations) or competing effects. The secondary covariates included baseline reports of alcohol status, smoking status, sleep pattern (derived using morning chronotype, sleep duration, insomnia, snoring, and daytime sleepiness) [45], diet pattern (from the intake of fruits, vegetables, fish, red meat, and processed meat) [46], self-rated health, employment status, and Townsend deprivation index (categorised into quintiles) [47]. These variables are most likely potential confounders (of the exposure–outcome associations), mediators (of the effect modifier-outcome associations), or competing effects.

The physical activity data had higher rates of missing values than other covariates. We used metabolic equivalent of task (MET) mins/week spent in moderate/vigorous physical activity in a sub-sample representing 80% of the full sample.

### Additional variables

Because differences in mortality or disease risk between groups (defined according to child and adult body weight) may be partly due to differences in adult body composition, we also used in descriptive analyses fat mass and trunk fat mass measures from Tanita BC418MA machines. These data were available for all participants in our sample.

### Statistical analysis

All analyses were stratified by sex in line with the Sex and Gender Equity in Research Guidelines. Supplementary Table 1 provides an overview of the number of participants included in each analysis.

The following nine groups were created using adulthood weight status and comparative weight at 10 years:

1. Normal weight_adult_, average_child_
2. Normal weight_adult_, thinner_child_
3. Normal weight_adult_, plumper_child_
4. Overweight_adult_, average_child_
5. Overweight_adult_, thinner_child_
6. Overweight_adult_, plumper_child_
7. Obesity_adult_, average_child_
8. Obesity_adult_, thinner_child_
9. Obesity_adult_, plumper_child_

Descriptive statistics were produced overall and for each of the nine groups. Comparative weight at 10 years was tabulated against adulthood weight status. For each adult weight status group, each comparative weight at 10 years group, and each of the nine groups 1) median and IQR values of BMI, fat mass, percent fat, trunk fat mass, and percent trunk fat were computed and 2) the number of participants, deaths, CVD events, and obesity-related cancer events were computed.

We used Cox proportional hazards regression, with age (scaled in years) as the underlying time scale. To help address the impact of reverse causality, the first two years of follow-up and any events within this period were excluded. The associations of comparative body weight at 10 years and, separately, adult weight status with each outcome were estimated. The primary models specifying a full factorial of these two variables (i.e., main terms for each variable and all interactions) were then developed. Likelihood-ratio tests were used to assess the difference between these models and equivalent models that did not include the interaction terms. The models with interactions were then used to obtain estimates of 1) the stratum-specific association of adult overweight or obesity versus normal weight with the outcomes in each comparative weight at 10 years group and 2) how the associations in #1 differed between the comparative weight at 10 years groups (i.e., the interactions). In Cox proportional hazards models, these interaction terms are equal to the ratio between the two stratum-specific estimates (e.g., estimate for obesity if plumper/estimate for obesity if average weight). Because interactions in Cox proportional hazards test departure from multiplicativity, we computed Relative Excess Risk due to Interaction (RERI) estimates to test departure from additivity [48]. The models were also re-parametrised to obtain hazard ratio (HR) estimates for eight of the nine groups, leaving the Normal weight_adult_, average_child_ group out as a referent. A figure was produced to illustrate these results. The main models included adjustment for age, ethnicity, relative age voice break (males) or age at menarche (females), and comparative height at age 10 years. A second set of models additionally adjusted for alcohol status, smoking status, sleep pattern, diet pattern, self-rated health, employment status, and Townsend index quintile. And, in the sub-sample, a third set further adjusted for MET mins/week for moderate/vigorous physical activity.

To triangulate results using an approach that better accounts for confounding [29, 30], the main models (with the same covariate adjustments) were repeated replacing childhood comparative body weight with childhood PRS categories and adulthood weight status with adult PRS categories. We also repeated the main models only replacing childhood comparative body weight with childhood PRS categories (i.e., keeping the exposure as measured adulthood weight status).

In part because the categorisation of adulthood BMI and the PRSs results in a loss of statistical power, we used them as continuous variables and examined 1) adult BMI – comparative child body size interactions, 2) adult PRS – child PRS interactions, and 3) adulthood weight status – child PRS interactions. These models included the same covariates as the main models.

Finally, as sensitivity analyses, we re-ran our main models after excluding participants with an ethnicity other than “White”.

For all models, the proportional hazard assumption was investigated graphically and tested using Schoenfeld residuals [49]. There was no evidence that this assumption was violated.

## Results

As shown in Table 1, less than 5% of the sample had an ethnicity other than “White”. 6% of men and 24% of women were living with obesity. In both sexes, the largest proportion of adults reported being about average weight at 10 years, followed by thinner, and then plumper. Adults with obesity were more likely to have reported being a plumper child than adults with normal weight (Table 2). Descriptive statistics for each of the nine groups, including for body composition and the number of deaths/events, are shown in Supplementary Tables 2–6.

**Table 1:** Description of the study sample.

		Males (*N* = 191,181)	Females (*N* = 242,806)
All-cause mortality			
Yes	*N* (%)	18,830 (9.9)	14,605 (6.0)
No	*N* (%)	171,164 (90.1)	227,462 (94.0)
Follow-up period (years)	Mean (SD)	13.4 (1.9)	13.6 (1.6)
Person years of follow-up	Count	2,549,159	3,299,232
Dropped (event within first two years of follow-up)	*N* (%)	1187 (0.6)	739 (0.3)
Incident CVD			
Yes	*N* (%)	66,027 (37.0)	65,347 (28.2)
No	*N* (%)	112,260 (63.0)	166,143 (71.8)
Follow-up period (years)	Mean (SD)	12.0 (4.0)	12.7 (3.7)
Person years of follow-up	Count	2,132,735	2,930,124
Dropped (event within first two years of follow-up)	*N* (%)	12,894 (6.7)	11,316 (4.7)
Incident obesity-related cancer			
Yes	*N* (%)	4803 (2.5)	5957 (2.5)
No	*N* (%)	185,746 (97.5)	235,985 (97.5)
Follow-up period (years)	Mean (SD)	13.0 (1.3)	13.0 (1.3)
Person years of follow-up	Count	2,475,450	3,146,550
Dropped (event within first two years of follow-up)	*N* (%)	632 (0.3)	864 (0.4)
BMI	Median (IQR)	27.3 (25.0, 30.0)	26.1 (23.5, 29.7)
Weight status			
Normal weight	*N* (%)	46,473 (24.3)	94,569 (39.0)
Overweight	N (%)	95,859 (50.1)	90,765 (37.4)
Obesity	N (%)	48,849 (25.5)	57,472 (23.7)
Comparative body weight at age 10 years			
About average weight	*N* (%)	98,240 (51.4)	123,031 (50.7)
Thinner	*N* (%)	66,791 (34.9)	76,761 (31.6)
Plumper	*N* (%)	26,150 (13.7)	43,014 (17.7)
Age	Mean (SD)	56.7 (8.2)	56.3 (7.9)
Ethnicity			
White	*N* (%)	183,416 (95.9)	231,707 (95.4)
Mixed	*N* (%)	834 (0.4)	1565 (0.6)
Asian	*N* (%)	3650 (1.9)	4109 (1.7)
Black	*N* (%)	2025 (1.1)	3517 (1.5)
Other	*N* (%)	1256 (0.7)	1908 (0.8)
Relative age voice broke			
Average	*N* (%)	171,374 (89.6)	–
Younger	*N* (%)	8396 (4.4)	–
Older	*N* (%)	11,411 (6.0)	–
Age of menarche	Mean (SD)	–	13.0 (1.6)
Comparative height at age 10 years			
About average height	*N* (%)	105,658 (55.3)	129,161 (53.2)
Shorter	*N* (%)	36,903 (19.3)	51,467 (21.2)
Taller	*N* (%)	48,620 (25.4)	62,178 (25.6)
Alcohol status			
Never	*N* (%)	4243 (2.2)	12,759 (5.3)
Previous	*N* (%)	6127 (3.2)	8525 (3.5)
Current	*N* (%)	180,811 (94.6)	221,522 (91.2)
Smoking status			
Never	*N* (%)	93,942 (49.1)	144,120 (59.4)
Previous	*N* (%)	74,678 (39.1)	77,605 (32.0)
Current	*N* (%)	22,561 (11.8)	21,081 (8.7)
Sleep pattern			
Healthy	*N* (%)	50,588 (26.5)	72,082 (29.7)
Intermediate	*N* (%)	116,419 (60.9)	146,172 (60.2)
Poor	*N* (%)	24,174 (12.6)	24,552 (10.1)
Diet pattern			
Good	*N* (%)	19,033 (10.0)	39,471 (16.3)
Reasonable	*N* (%)	130,897 (68.5)	180,592 (74.4)
Poor	*N* (%)	41,251 (21.6)	22,743 (9.4)
Self-rated health			
Excellent	*N* (%)	31,511 (16.5)	42,611 (17.6)
Good	*N* (%)	109,211 (57.1)	145,712 (60.0)
Fair	*N* (%)	41,755 (21.9)	45,670 (18.8)
Poor	*N* (%)	8684 (4.5)	8813 (3.6)
Employment status			
Employed or self-employed	*N* (%)	119,361 (62.4)	135,864 (56.0)
Retired	*N* (%)	59,659 (31.2)	85,312 (35.1)
Unemployed or other	*N* (%)	12,161 (6.4)	21,630 (8.9)
Physical activity			
MET mins/week in moderate/vigorous	Median (IQR)	960 (240, 2,400)	880 (240, 2,040)
Missing data	*N* (%)	29,218 (15.3)	58,641 (24.2)

**Table 2:** Tabulation of comparative body weight at age 10 years against weight status in adulthood.

	Males			Females		
	Comparative body weight at age 10 years			Comparative body weight at age 10 years		
	About average	Thinner	Plumper	About average	Thinner	Plumper
	*N*	*N*	*N*	*N*	*N*	*N*
Weight status in adulthood						
Normal weight	21,639	21,873	2,961	49,438	35,048	10,083
Overweight	52,182	32,422	11,255	47,314	27,606	15,845
Obesity	24,419	12,496	11,934	26,279	14,107	17,086
	Column %	Column %	Column %	Column %	Column %	Column %
Weight status in adulthood						
Normal weight	22.0	32.8	11.3	40.2	45.7	23.4
Overweight	53.1	48.5	43.0	38.5	36.0	36.8
Obesity	24.9	18.7	45.6	21.4	18.4	39.7
	Row %	Row %	Row %	Row %	Row %	Row %
Weight status in adulthood						
Normal weight	46.6	47.1	6.4	52.3	37.1	10.7
Overweight	54.4	33.8	11.7	52.1	30.4	17.5
Obesity	50.0	25.6	24.4	45.7	24.6	29.7

In both sexes, adulthood obesity (versus normal weight) was associated with higher hazards of all outcomes (Table 3). Plumper (versus about average) weight at 10 years was also associated with higher hazards of all outcomes. There was also evidence that the thinner group had higher hazards of all-cause mortality (females only) and incident CVD, but not obesity-related cancer.

**Table 3:** Separate associations of comparative body weight at age 10 years and adulthood weight status with all-cause mortality, cardiovascular disease, and obesity-related cancer.

	All-cause mortality				CVD			
	Males		Females		Males		Females	
	HR (95% CI)	*P*-value	HR (95% CI)	*P*-value	HR (95% CI)	*P*-value	HR (95% CI)	*P*-value
Weight status in adulthood^a^								
Normal weight (referent)	–		–		–		–	
Overweight	0.97 (0.93, 1.00)	0.079	0.99 (0.95, 1.03)	0.540	1.15 (1.13, 1.17)	<0.001	1.19 (1.16, 1.21)	<0.001
Obesity	1.15 (1.10, 1.20)	<0.001	1.15 (1.10, 1.20)	<0.001	1.41 (1.38, 1.44)	<0.001	1.44 (1.41, 1.47)	<0.001
Comparative body weight at age 10 years^b^								
About average weight (referent)	–		–		–		–	
Thinner	1.01 (0.98, 1.04)	0.629	1.08 (1.04, 1.13)	<0.001	1.03 (1.01, 1.05)	0.001	1.12 (1.10, 1.14)	<0.001
Plumper	1.18 (1.13, 1.23)	<0.001	1.27 (1.21, 1.32)	<0.001	1.12 (1.09, 1.14)	<0.001	1.13 (1.11, 1.16)	<0.001

	Obesity-related cancer			
	Males		Females	
	HR (95% CI)	*P*-value	HR (95% CI)	*P*-value
Weight status in adulthood^a^				
Normal weight (referent)	–		–	
Overweight	1.25 (1.16, 1.36)	<0.001	1.12 (1.06, 1.2)	0.001
Obesity	1.59 (1.46, 1.74)	<0.001	1.49 (1.39, 1.6)	<0.001
Comparative body weight at age 10 years^b^				
About average weight (referent)	–		–	
Thinner	0.95 (0.89, 1.01)	0.091	1.02 (0.96, 1.08)	0.589
Plumper	1.14 (1.05, 1.24)	0.002	1.14 (1.07, 1.22)	<0.001

^a^Models adjusted for age, ethnicity, relative age voice break (males) or age at menarche (females), comparative height at age 10 years, alcohol status, smoking status, sleep pattern, diet pattern, self-rated health, employment status, and Townsend index quintile.

^b^Models adjusted for age, ethnicity, relative age voice break (males) or age at menarche (females), and comparative height at age 10 years.

### All-cause mortality

In both sexes, the association of obesity (versus normal weight) with mortality was stronger in adults who reported being thinner at 10 years than in adults who reported being about average weight at 10 years (Table 4). For example, obesity was associated with a 1.28 (1.21, 1.35) times higher hazard of mortality in men who reported being an average weight child, but among men who reported being a thinner child this estimate was 1.63 (1.53, 1.75). The ratio between these two estimates was 1.28 (1.17, 1.40). The associated RERI estimate of 0.31 (0.21, 0.41) provides evidence of positive departure from additivity (i.e., the combined association of adulthood obesity and childhood thinness was larger than the sum of the two individual associations). After adjustment for adulthood variables, there was still evidence (for males but not females) that the association of obesity with mortality was stronger in the thinner child group (Supplementary Tables 7 and 8). In males, there was also evidence that the HR related to obesity was stronger in the plumper group than the about average comparative weight at 10 years group (Table 4), but after adjustment for adulthood variables this estimate became null (Supplementary Tables 7 and 8). The same pattern of effect modification as in the main models (i.e., not including the adult lifestyle covariates) was found when adult BMI was left as a continuous variable (Supplementary Table 9).

**Table 4:** Associations of adulthood overweight and obesity with all-cause mortality according to, and testing for effect modification by, comparative body weight at age 10 years: observational and genetic analyses.

	Males					
	Stratum-specific estimate		Interaction		RERI	
	HR (95% CI)	*P*-value	HR (95% CI)	*P*-value	Estimate (95% CI)	*P*-value
Overweight						
If average	0.95 (0.90, 1.00)	0.055	–		–	
If thinner	1.03 (0.97, 1.09)	0.304	1.09 (1.00, 1.17)	0.042	0.08 (0.00, 0.15)	0.037
If plumper	1.01 (0.88, 1.16)	0.901	1.06 (0.92, 1.23)	0.430	0.06 (−0.09, 0.21)	0.463
Obesity						
If average	1.28 (1.21, 1.35)	<0.001	–		–	
If thinner	1.63 (1.53, 1.74)	<0.001	1.28 (1.17, 1.40)	<0.001	0.31 (0.21, 0.41)	<0.001
If plumper	1.45 (1.26, 1.66)	<0.001	1.13 (0.98, 1.31)	0.094	0.18 (0.02, 0.34)	0.027
LRT vs model without interactions (*p* < 0.001)						
Overweight (using adult PRS)						
If average (using child PRS)	1.05 (1.00, 1.11)	0.054	–		–	
If thinner (using child PRS)	1.07 (1.01, 1.13)	0.016	1.02 (0.94, 1.10)	0.699	0.01 (−0.06, 0.09)	0.724
If plumper (using child PRS)	1.07 (0.94, 1.22)	0.332	1.01 (0.88, 1.17)	0.860	0.02 (−0.13, 0.17)	0.831
Obesity (using adult PRS)						
If average (using child PRS)	1.14 (1.08, 1.21)	<0.001	–		–	
If thinner (using child PRS)	1.15 (1.06, 1.23)	<0.001	1.00 (0.91, 1.10)	0.965	0.00 (−0.10, 0.10)	0.962
If plumper (using child PRS)	1.17 (1.02, 1.33)	0.024	1.02 (0.88, 1.18)	0.795	0.03 (−0.13, 0.18)	0.728
LRT vs model without interactions (p = 0.992)						

	Females					
	Stratum-specific estimate		Interaction		RERI	
	HR (95% CI)	*P*-value	HR (95% CI)	*P*-value	Estimate (95% CI)	*P*-value
Overweight						
If average	1.04 (0.98, 1.10)	0.187	–		–	
If thinner	1.10 (1.03, 1.18)	0.005	1.06 (0.97, 1.16)	0.180	0.06 (−0.03, 0.15)	0.161
If plumper	0.97 (0.87, 1.07)	0.525	0.93 (0.83, 1.05)	0.238	−0.08 (−0.22, 0.06)	0.268
Obesity						
If average	1.38 (1.30, 1.47)	<0.001	–		–	
If thinner	1.60 (1.49, 1.72)	<0.001	1.16 (1.06, 1.27)	0.002	0.24 (0.11, 0.36)	<0.001
If plumper	1.32 (1.20, 1.46)	<0.001	0.96 (0.85, 1.07)	0.464	0.02 (−0.13, 0.18)	0.765
LRT vs model without interactions (*p* = 0.010)						
Overweight (using adult PRS)						
If average (using child PRS)	1.06 (1.01, 1.12)	0.025	–		–	
If thinner (using child PRS)	1.08 (1.01, 1.15)	0.028	1.01 (0.93, 1.10)	0.800	0.01 (−0.08, 0.10)	0.781
If plumper (using child PRS)	0.98 (0.89, 1.09)	0.739	0.92 (0.82, 1.04)	0.181	−0.08 (−0.21, 0.04)	0.198
Obesity (using adult PRS)						
If average (using child PRS)	1.18 (1.11, 1.25)	<0.001	–		–	
If thinner (using child PRS)	1.12 (1.03, 1.23)	0.011	0.95 (0.85, 1.06)	0.370	−0.06 (−0.18, 0.06)	0.365
If plumper (using child PRS)	1.13 (1.02, 1.25)	0.021	0.96 (0.85, 1.08)	0.450	−0.04 (−0.18, 0.09)	0.536
LRT vs model without interactions (*p* = 0.480)						

Referent: normal weight in adulthood.

Models adjusted for age, ethnicity, relative age voice break (males) or age at menarche (females), and comparative height at age 10 years.

*LRT* likelihood-ratio test, *PRS* polygenic risk score, *RERI* relative excess risk due to interaction.

**Fig. 1:**
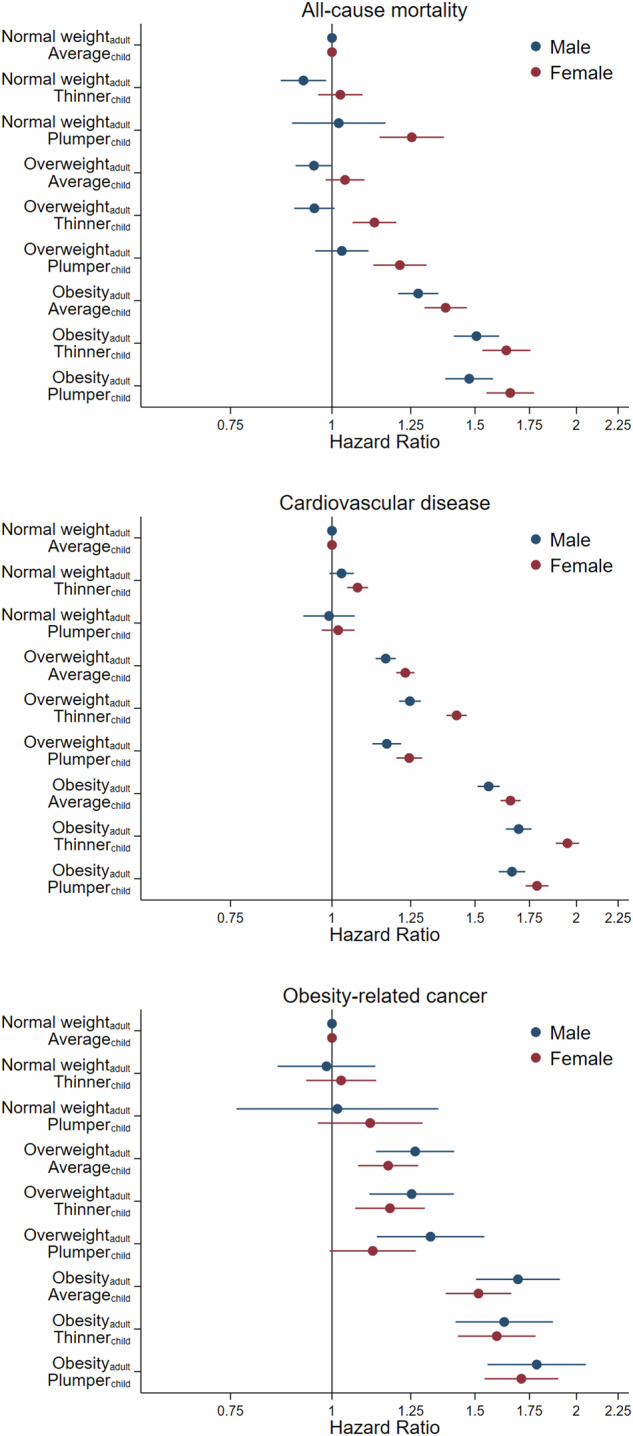
Hazard ratios for all-cause mortality, cardiovascular disease, and obesity-related cancer for each adulthood weight status and comparative body weight at 10 years group. Referent: Normal weight adult, about average weight child. Models adjusted for age, ethnicity, relative age voice break (males) or age at menarche (females), and comparative height at age 10 years.

Using the re-parametrised main models (see methods), Fig. 1 shows the HRs for each child-adult body size group relative to normal weight_adult_, average_child_. The highest HRs were seen in the 1) obesity_adult_, thinner_child_ and 2) obesity_adult_, plumper_child_ groups.

In genetic analyses, there was no evidence that the associations of overweight or obesity (according to the adult PRS) with all-cause mortality differed according to childhood body size (according to the child PRS) (Table 4). Additional analyses using the PRSs also produced mainly null effect modification results. One exception was that, for males, we found evidence that the association of overweight with mortality was weaker in the thinner group (according to the child PRS) (Supplementary Table 10). Relatedly, there was evidence of a positive interaction between adult overweight and the child PRS (Supplementary Table 11).

As shown in Supplementary Table 12, the main results were very similar when the sample was restricted to participants of white ethnicity.

### Incident cardiovascular disease

Results were similar to those for all-cause mortality (Table 5). In both sexes, the associations of obesity (versus normal weight) with incident CVD were stronger in adults who reported being thinner at 10 years, and in adults who reported being plumper at 10 years, compared to adults who reported being about average weight at 10 years. The HRs related to overweight were also stronger in the thinner group, but not the plumper group. Adjustment for adulthood variables attenuated all interaction estimates to the null in males but much less so in females (Supplementary Tables 13 and 14). Even in the fully adjusted models (including physical activity), the associations, in females, of adulthood overweight and obesity with CVD were larger in the thinner than average comparative childhood weight group. In models using adult BMI as a continuous variable, there was also evidence of stronger associations in the thinner group (Supplementary Table 15).

**Table 5:** Associations of adulthood overweight and obesity with incident cardiovascular disease according to, and testing for effect modification by, comparative body weight at age 10 years: observational and genetic analyses.

	Males					
	Stratum-specific estimate		Interaction		RERI	
	HR (95% CI)	*P*-value	HR (95% CI)	*P*-value	Estimate (95% CI)	*P*-value
Overweight						
If average	1.16 (1.13, 1.20)	<0.001	–		–	
If thinner	1.21 (1.18, 1.25)	<0.001	1.04 (1.00, 1.09)	0.054	0.05 (0.01, 0.10)	0.018
If plumper	1.18 (1.09, 1.27)	<0.001	1.01 (0.93, 1.10)	0.785	0.01 (−0.07, 0.09)	0.813
Obesity						
If average	1.56 (1.51, 1.61)	<0.001	–		–	
If thinner	1.65 (1.59, 1.71)	<0.001	1.06 (1.01, 1.11)	0.018	0.11 (0.05, 0.18)	0.001
If plumper	1.68 (1.56, 1.81)	<0.001	1.08 (0.99, 1.17)	0.071	0.11 (0.02, 0.20)	0.014
LRT vs model without interactions (*p* = 0.018)						
Overweight (using adult PRS)						
If average (using child PRS)	1.03 (1.00, 1.06)	0.024	–		–	
If thinner (using child PRS)	1.05 (1.02, 1.08)	<0.001	1.02 (0.98, 1.06)	0.353	0.02 (−0.02, 0.06)	0.346
If plumper (using child PRS)	1.02 (0.95, 1.09)	0.610	0.99 (0.91, 1.06)	0.710	−0.01 (−0.09, 0.06)	0.721
Obesity (using adult PRS)						
If average (using child PRS)	1.08 (1.05, 1.12)	<0.001	–		–	
If thinner (using child PRS)	1.10 (1.05, 1.14)	<0.001	1.01 (0.96, 1.07)	0.600	0.01 (−0.04, 0.07)	0.602
If plumper (using child PRS)	1.06 (0.99, 1.14)	0.102	0.98 (0.91, 1.06)	0.615	−0.02 (−0.10, 0.06)	0.614
LRT vs model without interactions (*p* = 0.847)						

	Females					
	Stratum-specific estimate		Interaction		RERI	
	HR (95% CI)	*P*-value	HR (95% CI)	*P*-value	Estimate (95% CI)	*P*-value
Overweight						
If average	1.23 (1.20, 1.26)	<0.001	–		–	
If thinner	1.32 (1.28, 1.37)	<0.001	1.08 (1.03, 1.12)	<0.001	0.11 (0.07, 0.16)	<0.001
If plumper	1.22 (1.16, 1.29)	<0.001	0.99 (0.94, 1.05)	0.826	0.00 (−0.07, 0.06)	0.991
Obesity						
If average	1.66 (1.61, 1.71)	<0.001	–		–	
If thinner	1.81 (1.75, 1.88)	<0.001	1.09 (1.05, 1.14)	<0.001	0.22 (0.15, 0.28)	<0.001
If plumper	1.76 (1.67, 1.85)	<0.001	1.06 (1.00, 1.12)	0.049	0.11 (0.04, 0.18)	0.004
LRT vs model without interactions (*p* < 0.001)						
Overweight (using adult PRS)						
If average (using child PRS)	1.02 (1.00, 1.05)	0.061	–		–	
If thinner (using child PRS)	1.03 (1.00, 1.07)	0.032	1.01 (0.97, 1.05)	0.633	0.01 (−0.03, 0.05)	0.644
If plumper (using child PRS)	0.99 (0.94, 1.04)	0.602	0.96 (0.91, 1.02)	0.188	−0.04 (−0.09, 0.02)	0.188
Obesity (using adult PRS)						
If average (using child PRS)	1.07 (1.04, 1.10)	<0.001	–		–	
If thinner (using child PRS)	1.08 (1.04, 1.13)	<0.001	1.01 (0.96, 1.06)	0.684	0.01 (−0.04, 0.06)	0.717
If plumper (using child PRS)	1.06 (1.01, 1.12)	0.017	0.99 (0.94, 1.05)	0.759	−0.01 (−0.07, 0.05)	0.759
LRT vs model without interactions (*p* = 0.569)						

Referent: normal weight in adulthood.

Models adjusted for age, ethnicity, relative age voice break (males) or age at menarche (females), and comparative height at age 10 years.

*LRT* likelihood-ratio test, *PRS* polygenic risk score, *RERI* relative excess risk due to interaction.

Figure 1 clearly illustrates 1) that out of the three adulthood groups with overweight, the highest HRs were observed in the overweight_adult_, thinner_child_ group and 2) that out of the three adulthood obesity groups, the highest HRs were observed in the obesity_adult_, thinner_child_ group.

In all genetic analyses, there was no evidence of effect modification (Table 5 and Supplementary Tables 16 & 17).

As shown in Supplementary Table 18, the main results were very similar when the sample was restricted to participants of white ethnicity.

### Incident obesity-related cancer

In all models, there was no evidence that the association of obesity (or overweight) with incident cancer differed between the three comparative weight at 10 years groups (Table 6, Supplementary Tables 19 and 20). Further, in models using adult BMI as a continuous variable, all interaction terms were null (Supplementary Table 21).

**Table 6:** Associations of adulthood overweight and obesity with incident obesity-related cancer according to, and testing for effect modification by, comparative body weight at age 10 years: observational and genetic analyses.

	Males					
	Stratum-specific estimate		Interaction		RERI	
	HR (95% CI)	*P*-value	HR (95% CI)	*P*-value	Estimate (95% CI)	*P*-value
Overweight						
If average	1.27 (1.13, 1.41)	<0.001	–		–	
If thinner	1.27 (1.13, 1.44)	<0.001	1.01 (0.85, 1.19)	0.946	0.00 (−0.17, 0.18)	0.980
If plumper	1.30 (0.97, 1.75)	0.078	1.03 (0.75, 1.41)	0.860	0.04 (−0.30, 0.37)	0.822
Obesity						
If average	1.69 (1.50, 1.91)	<0.001	–		–	
If thinner	1.65 (1.44, 1.90)	<0.001	0.98 (0.81, 1.17)	0.802	−0.05 (−0.29, 0.19)	0.679
If plumper	1.76 (1.32, 2.34)	<0.001	1.04 (0.76, 1.42)	0.812	0.08 (−0.28, 0.44)	0.673
LRT vs model without interactions (*p* = 0.992)						
Overweight (using adult PRS)						
If average (using child PRS)	1.14 (1.02, 1.27)	0.018	–		–	
If thinner (using child PRS)	1.08 (0.97, 1.21)	0.160	0.95 (0.81, 1.11)	0.513	−0.06 (−0.22, 0.11)	0.494
If plumper (using child PRS)	1.03 (0.80, 1.33)	0.814	0.91 (0.69, 1.19)	0.479	−0.10 (−0.42, 0.22)	0.535
Obesity (using adult PRS)						
If average (using child PRS)	1.27 (1.13, 1.43)	<0.001	–		–	
If thinner (using child PRS)	1.00 (0.85, 1.17)	0.974	0.78 (0.64, 0.95)	0.015	−0.28 (−0.49, −0.06)	0.013
If plumper (using child PRS)	1.03 (0.80, 1.33)	0.830	0.81 (0.61, 1.07)	0.139	−0.24 (−0.57, 0.09)	0.159
LRT vs model without interactions (p = 0.104)						

	Females					
	Stratum-specific estimate		Interaction		RERI	
	HR (95% CI)	*P*-value	HR (95% CI)	*P*-value	Estimate (95% CI)	*P*-value
Overweight						
If average	1.17 (1.08, 1.28)	<0.001	–		–	
If thinner	1.15 (1.03, 1.28)	0.011	0.98 (0.85, 1.12)	0.763	-0.02 (−0.17, 0.13)	0.777
If plumper	1.01 (0.85, 1.19)	0.933	0.86 (0.71, 1.04)	0.117	−0.17 (−0.38, 0.05)	0.130
Obesity						
If average	1.51 (1.38, 1.66)	<0.001	–		–	
If thinner	1.55 (1.38, 1.75)	<0.001	1.03 (0.88, 1.19)	0.737	0.06 (−0.14, 0.26)	0.572
If plumper	1.53 (1.31, 1.80)	<0.001	1.01 (0.84, 1.22)	0.888	0.08 (−0.15, 0.32)	0.485
LRT vs model without interactions (p = 0.319)						
Overweight (using adult PRS)						
If average (using child PRS)	0.97 (0.89, 1.05)	0.471	–		–	
If thinner (using child PRS)	1.05 (0.95, 1.16)	0.310	1.09 (0.95, 1.24)	0.215	0.08 (−0.05, 0.21)	0.210
If plumper (using child PRS)	1.07 (0.91, 1.26)	0.434	1.10 (0.91, 1.33)	0.306	0.09 (−0.08, 0.27)	0.298
Obesity (using adult PRS)						
If average (using child PRS)	0.99 (0.90, 1.09)	0.855	–		–	
If thinner (using child PRS)	0.90 (0.77, 1.04)	0.144	0.90 (0.76, 1.08)	0.260	−0.10 (−0.26, 0.07)	0.263
If plumper (using child PRS)	1.17 (0.99, 1.37)	0.067	1.18 (0.97, 1.42)	0.093	0.16 (−0.02, 0.35)	0.088
LRT vs model without interactions (p = 0.073)						

Referent: normal weight in adulthood.

Models adjusted for age, ethnicity, relative age voice break (males) or age at menarche (females), and comparative height at age 10 years.

*LRT* likelihood-ratio test, *PRS* polygenic risk score, *RERI* relative excess risk due to interaction.

In genetic analyses, the association of adult obesity (according to the adult PRS) with incident obesity-related cancer was weaker in the thinner group than in the average group (according to the child PRS) for males (Table 6). A different, inconsistent pattern of results was found in the analysis using adulthood weight status and the child PRS categories (Supplementary Table 22), while analyses using continuous PRS(s) produced no evidence of effect modification (Supplementary Table 23).

As shown in Supplementary Table 24, the main results were very similar when the sample was restricted to participants of white ethnicity.

## Discussion

This paper provides the first evidence from a large-scale contemporary study that the same pattern of results for CVD, first reported by Abraham et al. in 1971 [12], might also be present for all-cause mortality. However, while the observational associations of obesity with all-cause mortality (and incident CVD) were stronger in adults who reported being a thinner or plumper than average child, analyses using genetic instruments were null and did not produce a similar pattern of effect modification results. Greater risks for all-cause mortality and incident CVD in adults with obesity who perceive themselves to have been a thinner or plumper than average child may, therefore, be due to confounding and/or recall bias.

Many papers have conducted multivariable Mendelian randomisation analysis to investigate the extent to which childhood body size affects disease risk independent of adulthood BMI [31, 42–44, 50, 51]. We are not, however, aware of any studies that have conducted factorial Mendelian randomisation (analogous to a factorial randomized controlled trial) to investigate the interactive associations of childhood body weight and adulthood BMI with disease outcomes. Our paper is the first to examine interactions between PRSs for comparative childhood body weight and adulthood BMI in relation to all-cause mortality and incident CVD and obesity-related cancer. While these analyses benefited from the core underlying principle of Mendelian randomization (i.e., random allocation of parental alleles to zygotes at meiosis), producing results that are robust to reverse causation and considerably less prone to confounding [29, 30], we did not derive formal Mendelian randomisation estimates. This is because standard Mendelian randomisation approaches using Cox proportional hazard models can induce significant bias [52]. Overall, our genetic analyses failed to confirm the observational results for all-cause mortality and incident CVD. This may, however, reflect a lack of efficiency in testing interactions between PRSs. Rees et al have demonstrated how a new method that uses all available genetic variants and their interactions as instrumental variables substantially increases efficiency [53]. When this method is developed further (e.g., for survival outcomes), future research may be able to address our research question using advanced, formal Mendelian randomisation analysis.

Despite our genetic results not supporting our observational results, descriptive statistics did show higher levels of whole body and trunk fat mass in the obesity_adult_, thinner_child_ and obesity_adult_, plumper_child_ groups than the obesity_adult_, average_child_ group. Differences in adulthood adiposity might therefore partly explain the pattern of effect modification we observed. Alternatively, if the effect modification we reported in observational analyses does not reflect childhood body weight causally affecting the relationship of excess adiposity with mortality and CVD risk, as suggested by our PRS analyses, then it may reflect fundamental differences (e.g., behavioural and psychological) between adults who have the same weight status, but different perceptions of their childhood body weight. We found consistent evidence of this in our descriptive analyses. For example, adults with obesity who reported being plumper at 10 years were more likely to be a current smoker than adults with obesity who reported being about average weight at 10 years (12.5% vs 10.2% for males). Such differences are likely to reflect underlying inequalities. Socioeconomic position tracks across the life course [54], with more disadvantaged socioeconomic position associated with greater risks of both childhood obesity and thinness [55, 56], as well as unhealthy behaviours (e.g., smoking) in adulthood [57]. These known patterns of inequalities help explain why the three groups within each adulthood weight status category differed with respect to covariates. As a result, in our analyses, most interaction estimates attenuated upon adjustment for adulthood lifestyle behaviours.

Both weight status in adulthood and comparative body weight at 10 years were associated with obesity-related cancer in the expected direction, but we observed no strong or consistent evidence of effect modification in our main observational analyses. The genetic analyses provided some evidence of effect modification, but this was inconsistent between sexes, models (i.e., including only the child PRS or including both child and adult PRSs), and sometimes in different directions for adulthood overweight and obesity. Despite the large sample size, the number of adults who developed obesity-related cancer was relatively small (*N* = 10,760, 2.5% of sample), which increases the risk of spurious results [58]. Our genetic results for obesity-related cancer, therefore, need to be interpreted with caution.

Only one other UK Biobank study has been published on the same topic as the present paper. Carrasquilla et al investigated child-to-adult body size trajectories and risk of type 2 diabetes and CVD [59], using outcomes that were algorithmically defined by the UK Biobank [60, 61]. However, partly because the algorithmically defined CVD outcome in UK Biobank only includes myocardial infarction and stroke, only 5% of the sample in the Carrasquilla et al paper had incident CVD, compared to 30% in our sample. Subsequently, Carrasquilla et al did not have the numbers necessary to stratify their main analyses by sex or to consider standard, clinically relevant adulthood weight status groups (e.g., normal weight, overweight, obesity). Instead, they created three adult body size groups (low, average, high), each of which had the same N as the corresponding childhood body size group (thinner, average, plumper). They then created the nine groups that combine child and adult body size and represent trajectories, and conducted analyses to produce estimates equivalent to what we show in our Fig. 1. For CVD, their key finding was that disease risk was comparable in the three groups with high adulthood BMI, suggesting that “CVD risk was determined by adult body size, irrespective of childhood body size”. Effect modification of the association of high adulthood BMI with incident CVD by comparative body weight at 10 years was not, however, explicitly tested.

Our non-genetic results are very different to those of Carrasquilla et al and tie in with the wider literature suggesting that the greatest risk of CVD is observed in individuals with low or high childhood BMI followed by high adulthood BMI [18]. If anything, the later of the two may be more harmful to health. For example, in a population-based cohort study of 37,672 Swedish men, Ohlsson et al reported a higher hazard ratio for cardiovascular mortality in boys who were normal weight at 8 years but overweight at 20 years (HR 2.39; 1.86, 3.09) than in boys who were overweight at both ages (HR 1.85; 1.28, 2.67) [62]. One explanation for these findings is that adolescent and early adulthood BMI gains are more strongly related (than childhood BMI gains) to increases in visceral adiposity [63, 64], which we know plays an important role in CVD aetiology [65]. There is also evidence that lower childhood BMI itself is deleterious. The study of Hawkes et al, for example, provides genetic evidence that lower childhood BMI is associated with worse intermediate diabetes traits (which are also involved in the pathophysiology of CVD) after accounting for adulthood BMI [66].

The key strength of our study is the large number of participants with body size data in childhood and adulthood, combined with mortality and incident disease outcomes. While comparative childhood body weight at 10 years being self-reported could be seen as a limitation, it is also a strength because it reflects the type of information that could be easily obtained in real world settings and studies of only adults. We know that (on average) adults with higher BMI values tend to underreport their current weight [67, 68], but we do not know how comparative weight at 10 years might suffer from recall bias. In the absence of any recall bias, one would expect adults with obesity to be more likely than adults who are overweight or normal weight to report being a plumper than average child [69], and this is what we observed. There were several key early life variables that ideally we would have treated as confounders, but unfortunately these were either not assessed (e.g., parental BMI and socioeconomic position) or had large amounts of missing data (e.g., birth weight). Despite the large sample size, we did not have the numbers necessary to investigate cause-specific mortality or to perform analyses stratified by ethnic group. More generally, participants in the UK Biobank study are not representative of the general population; there is a “healthy volunteer” selection bias which has been shown to bias associations [70–74]. This does not, however, necessarily mean our estimates are biased [75]. It has even been argued that representativeness should be avoided in observational cohort studies [76]. Distinguishing interactions (such as those tested in our paper) from non-linear associations is difficult [77], and we acknowledge that non-linear associations of continuous body weight at 10 years (if available) and/or adulthood BMI with an outcome may produce or masquerade as a interaction. The similarity of a model with an interaction (e.g., child weight X adult BMI) and a model capturing a non-linear association (e.g., a quadratic term for adult BMI) ultimately depends on the correlation between the two exposures. The point biserial correlations between comparative child body size and adult BMI were low in our sample (<0.3) suggesting that the interactions we observed are unlikely to be nonlinear associations in disguise. Further, the likelihood ratio tests we present empirically compare models with interactions against models with non-linear terms (using categories). This is aligned with the approach recommended by Belzak and Bauer [77]. We also acknowledge that, regardless of potential non-linearity, categorisation of exposures can lead to spurious interactions [78]. This is why we also ran models using continuous variables; these results support our main findings. Finally, we did not consider breast cancer in our analyses because these would have to be separate models, stratified by menopause status. This is a substantial standalone topic and the goal of future planned research.

## Conclusions

In conclusion, while other publications have examined differences in disease risk across “trajectory” groups and interpreted their findings through a prospective lens, the focus of our paper was on effect modification (requiring explicit testing of interactions that is often not performed in other studies) with interpretation through a retrospective lens [7–11]. Approaching the research in this way makes intuitive sense given that comparative body size was self-reported by adults. The association of obesity with all-cause mortality and incident CVD was stronger in adults who reported being a thinner or plumper than average child, but analyses using genetic instruments did not produce a similar pattern of effect modification results. Research is therefore needed to understand the other factors (e.g., behavioural, psychological, and socioeconomic) that might explain why adults living with obesity, who perceive themselves to have been a thinner or plumper than average child, have the greatest mortality and CVD risks.

## Supplementary information

SUPPLEMENTAL MATERIAL

## Data Availability

UK Biobank data are available to any bona fide researcher to conduct health-related research that is in the public interest at http://www.ukbiobank.ac.uk/using-the-resource/.

## Code availability

Available from the first author upon request.
