# Supplementary material for: Are associations of adulthood overweight and obesity with all-cause mortality, cardiovascular disease, and obesity-related cancer modified by comparative body weight at age 10 years in the UK Biobank study?
Source: Int J Obes (Lond). 2025 Jan 23;49(5):902–14. doi: 10.1038/s41366-025-01718-4 (PMC12095051; doi:10.1038/s41366-025-01718-4)
Supplement: Supplementary file 1 — SUPPLEMENTAL MATERIAL [file 41366_2025_1718_MOESM1_ESM.docx]

SUPPLEMENTARY MATERIAL

**Are associations of adulthood overweight and obesity with all-cause mortality, cardiovascular disease, and obesity-related cancer modified by comparative body weight at age 10 years in the UK Biobank study?**

William Johnson, Tom Norris, Natalie Pearson, Emily S Petherick, James A King, Scott A Willis, Rebecca Hardy, Susan Paudel, Emma Haycraft, Jennifer L Baker, Mark Hamer, David J Stensel, Kate Tilling, Tom G Richardson

Supplementary Table 1. Overview of the study numbers

|  | Male | Female |
| --- | --- | --- |
| Sample | 191,181 | 242,806 |
| All-cause mortality | 189,994 | 242,067 |
| Incident CVD | 178,287 | 231,490 |
| Incident obesity-related cancer | 190,549 | 241,942 |
| Sub-sample with physical activity data | 161,936 | 184,165 |
| All-cause mortality | 161,014 | 183,633 |
| Incident CVD | 151,441 | 176,088 |
| Incident obesity-related cancer | 161,429 | 183,527 |
| Sub-sample with polygenic risk scores | 187,919 | 235,160 |
| All-cause mortality | 186,762 | 234,457 |
| Incident CVD | 175,255 | 224,207 |
| Incident obesity-related cancer | 187,735 | 234,323 |

Supplementary Table 2. Description of the study sample, stratified by comparative body weight at age 10 years and adulthood weight status (Males)

|  |  | Males | | | | | | | | |
| --- | --- | --- | --- | --- | --- | --- | --- | --- | --- | --- |
|  |  | Child: about average | | | Child: thinner | | | Child: plumper | | |
|  |  | Adult:  normal weight | Adult:  overweight | Adult:  obesity | Adult:  normal weight | Adult:  overweight | Adult:  obesity | Adult:  normal weight | Adult:  overweight | Adult:  obesity |
| Age | Mean (SD) | 56.5 (8.4) | 57.0 (8.2) | 57.1 (7.9) | 55.9 (8.3) | 56.9 (8.1) | 57.4 (7.9) | 55.4 (8.1) | 56.1 (8.2) | 55.8 (7.9) |
| Ethnicity |  |  |  |  |  |  |  |  |  |  |
| White | N (%) | 20,677 (95.6) | 50,289 (96.4) | 23,517 (96.3) | 20,856 (95.4) | 30,932 (95.4) | 11,934 (95.5) | 2,839 (95.9) | 10,817 (96.1) | 11,555 (96.8) |
| Other | N (%) | 962 (4.5) | 1,893 (3.6) | 902 (3.7) | 1,017 (4.7) | 1,490 (4.6) | 562 (4.5) | 122 (4.1) | 438 (3.9) | 379 (3.2) |
| Relative age voice broke |  |  |  |  |  |  |  |  |  |  |
| Average | N (%) | 19,716 (91.1) | 47,906 (91.8) | 22,328 (91.4) | 18,629 (85.2) | 28,448 (87.7) | 11,127 (89.0) | 2,604 (87.9) | 10,046 (89.3) | 10,570 (88.6) |
| Younger | N (%) | 727 (3.4) | 2,114 (4.1) | 1,377 (5.6) | 678 (3.1) | 1,213 (3.7) | 623 (5.0) | 132 (4.5) | 640 (5.7) | 892 (7.5) |
| Older | N (%) | 1,196 (5.5) | 2,162 (4.1) | 714 (2.9) | 2,566 (11.7) | 2,761 (8.5) | 746 (6.0) | 225 (7.6) | 569 (5.1) | 472 (4.0) |
| Comparative height at age 10 years |  |  |  |  |  |  |  |  |  |  |
| About average height | N (%) | 13,781 (63.7) | 33,112 (63.5) | 15,493 (63.5) | 9,649 (44.1) | 14,378 (44.4) | 5,764 (46.1) | 1,543 (52.1) | 5,889 (52.3) | 6,049 (50.7) |
| Shorter | N (%) | 3,250 (15.0) | 9,893 (15.1) | 3,380 (13.8) | 5,869 (26.8) | 9,052 (27.9) | 3,416 (27.3) | 517 (17.5) | 1,731 (15.4) | 1,795 (15.0) |
| Taller | N (%) | 4,608 (21.3) | 11,177 (21.4) | 5,546 (22.7) | 6,355 (29.1) | 8,992 (27.7) | 3,316 (26.5) | 901 (30.4) | 3,635 (32.3) | 4,090 (34.3) |
| Alcohol status |  |  |  |  |  |  |  |  |  |  |
| Never | N (%) | 531 (2.5) | 1,065 (2.0) | 583 (2.4) | 545 (2.5) | 665 (2.1) | 339 (2.7) | 59 (2.0) | 200 (1.8) | 256 (2.2) |
| Previous | N (%) | 703 (3.3) | 1,404 (2.7) | 889 (3.6) | 727 (3.3) | 1,013 (3.1) | 573 (4.6) | 103 (3.5) | 314 (2.8) | 401 (3.4) |
| Current | N (%) | 20,405 (94.3) | 49,713 (95.3) | 22,947 (94.0) | 20,601 (94.2) | 30,744 (94.8) | 11,584 (92.7) | 2,799 (94.5) | 10,741 (95.4) | 11,277 (94.5) |
| Smoking status |  |  |  |  |  |  |  |  |  |  |
| Never | N (%) | 11,953 (55.2) | 26,001 (49.8) | 10,653 (43.6) | 12,245 (56.0) | 15,506 (47.8) | 5,145 (41.2) | 1,597 (53.9) | 5,520 (49.0) | 5,322 (44.6) |
| Previous | N (%) | 6,369 (29.4) | 20,192 (38.7) | 11,288 (46.2) | 6,927 (31.7) | 13,558 (41.8) | 6,045 (48.4) | 910 (30.7) | 4,266 (37.9) | 5,123 (42.9) |
| Current | N (%) | 3,317 (15.3) | 5,989 (11.5) | 2,478 (10.2) | 2,701 (12.4) | 3,358 (10.4) | 1,306 (10.5) | 454 (15.3) | 1,469 (13.1) | 1,489 (12.5) |
| Sleep pattern |  |  |  |  |  |  |  |  |  |  |
| Healthy | N (%) | 7,230 (33.4) | 14,776 (28.3) | 5,187 (21.2) | 6,690 (30.6) | 7,905 (24.4) | 2,337 (18.7) | 1,011 (34.1) | 3,031 (26.9) | 2,421 (20.3) |
| Intermediate | N (%) | 12,622 (58.3) | 31,835 (61.0) | 15,193 (62.2) | 13,034 (59.6) | 20,173 (62.2) | 7,632 (61.1) | 1,710 (57.8) | 6,841 (60.8) | 7,379 (61.8) |
| Poor | N (%) | 1,787 (8.3) | 5,571 (10.7) | 4,039 (16.5) | 2,149 (9.8) | 4,344 (13.4) | 2,527 (20.2) | 240 (8.1) | 1,383 (12.3) | 2,134 (17.9) |
| Diet pattern |  |  |  |  |  |  |  |  |  |  |
| Good | N (%) | 2,503 (11.6) | 5,430 (10.4) | 2,243 (9.2) | 2,114 (9.7) | 3,053 (9.4) | 1,113 (8.9) | 342 (11.6) | 1,179 (10.5) | 1056 (8.9) |
| Reasonable | N (%) | 14,828 (68.5) | 36,138 (69.3) | 16,518 (67.6) | 14,876 (68.0) | 22,060 (68.0) | 8,369 (67.0) | 2,085 (70.4) | 7,900 (70.2) | 8,123 (68.1) |
| Poor | N (%) | 4,308 (19.9) | 10,614 (20.3) | 5,658 (23.2) | 4,883 (22.3) | 7,309 (22.5) | 3,014 (24.1) | 534 (18.0) | 2,176 (19.3) | 2,755 (23.1) |
| Self-rated health |  |  |  |  |  |  |  |  |  |  |
| Excellent | N (%) | 5,489 (25.4) | 9,657 (18.5) | 1,962 (8) | 5,251 (24.0) | 5,018 (15.5) | 741 (5.9) | 754 (25.5) | 1,863 (16.6) | 776 (6.5) |
| Good | N (%) | 12,384 (57.2) | 31,979 (61.3) | 13,044 (53.4) | 12,636 (57.8) | 19,170 (59.1) | 5,739 (45.9) | 1,675 (56.6) | 6,693 (59.5) | 5,891 (49.4) |
| Fair | N (%) | 3,161 (14.6) | 9,226 (17.7) | 7,684 (31.5) | 3,285 (15.0) | 6,988 (21.6) | 4,583 (36.7) | 436 (14.7) | 2,280 (20.3) | 4,132 (34.6) |
| Poor | N (%) | 605 (2.8) | 1,320 (2.5) | 1,729 (7.1) | 701 (3.2) | 1,246 (3.8) | 1,433 (11.5) | 96 (3.2) | 419 (3.7) | 1,135 (9.5) |
| Employment status |  |  |  |  |  |  |  |  |  |  |
| Employed or self-employed | N (%) | 13,255 (61.3) | 32,502 (62.3) | 14,949 (61.2) | 14,213 (65.0) | 20,325 (62.7) | 7,178 (57.4) | 1,959 (66.2) | 7,236 (64.3) | 7,744 (64.9) |
| Retired | N (%) | 6,994 (32.3) | 17,102 (32.8) | 7,543 (30.9) | 6,473 (29.6) | 10,276 (31.7) | 3,987 (31.9) | 813 (27.5) | 3,324 (29.5) | 3,147 (26.4) |
| Unemployed or other | N (%) | 1,390 (6.4) | 2,578 (4.9) | 1,927 (7.9) | 1,187 (5.4) | 1,821 (5.6) | 1,331 (10.7) | 189 (6.4) | 695 (6.2) | 1,043 (8.7) |

Supplementary Table 3. Description of the study sample, stratified by comparative body weight at age 10 years and adulthood weight status (Females)

|  |  | Females | | | | | | | | |
| --- | --- | --- | --- | --- | --- | --- | --- | --- | --- | --- |
|  |  | Child: about average | | | Child: thinner | | | Child: plumper | | |
|  |  | Adult:  normal weight | Adult:  overweight | Adult:  obesity | Adult:  normal weight | Adult:  overweight | Adult:  obesity | Adult:  normal weight | Adult:  overweight | Adult:  obesity |
| Age | Mean (SD) | 55.6 (8.1) | 57.2 (7.8) | 57.1 (7.7) | 55.0 (8.2) | 57.4 (7.9) | 57.6 (7.8) | 55.1 (7.9) | 56.0 (7.9) | 55.6 (7.8) |
| Ethnicity |  |  |  |  |  |  |  |  |  |  |
| White | N (%) | 47,828 (96.7) | 45,541 (96.3) | 24,936 (94.9) | 33,369 (95.2) | 25,953 (94.0) | 12,883 (91.3) | 9,777 (97.0) | 15,228 (96.1) | 16,192 (94.8) |
| Other | N (%) | 1,610 (3.3) | 1,773 (3.8) | 1,343 (5.1) | 1,679 (4.8) | 1,643 (6.0) | 1,224 (8.7) | 306 (3.0) | 617 (3.9) | 894 (5.2) |
| Age of menarche | Mean (SD) | 13.1 (1.5) | 12.8 (1.5) | 12.6 (1.6) | 13.5 (1.6) | 13.3 (1.6) | 13.0 (1.8) | 12.7 (1.6) | 12.6 (1.6) | 12.3 (1.7) |
| Comparative height at age 10 years |  |  |  |  |  |  |  |  |  |  |
| About average height | N (%) | 30,214 (61.1) | 28,694 (60.7) | 16,038 (61.0) | 14,868 (42.4) | 11,995 (43.5) | 6,416 (45.5) | 4,855 (48.2) | 7,702 (48.6) | 8,379 (49.0) |
| Shorter | N (%) | 8,234 (16.7) | 8,222 (17.4) | 4,572 (17.4) | 10,033 (28.6) | 8,001 (29.0) | 4,002 (28.4) | 1,905 (18.9) | 3,029 (19.1) | 3,469 (20.3) |
| Taller | N (%) | 10,990 (22.2) | 10,398 (22.0) | 5,669 (21.6) | 10,147 (29.0) | 7,610 (27.6) | 3,689 (26.2) | 3,323 (33.0) | 5,114 (32.3) | 5,238 (30.7) |
| Alcohol status |  |  |  |  |  |  |  |  |  |  |
| Never | N (%) | 2,028 (4.1) | 2,416 (5.1) | 1,875 (7.1) | 1,580 (4.5) | 1,583 (5.7) | 1,268 (9.0) | 364 (3.6) | 604 (3.8) | 1,041 (6.1) |
| Previous | N (%) | 1,402 (2.8) | 1,424 (3.0) | 1,186 (4.5) | 1,030 (2.9) | 1,030 (3.7) | 844 (6.0) | 360 (3.6) | 462 (2.9) | 787 (4.6) |
| Current | N (%) | 46,008 (93.1) | 43,474 (91.9) | 23,218 (88.4) | 32,438 (92.6) | 24,993 (90.5) | 11,995 (85.0) | 9,359 (92.8) | 14,779 (93.3) | 15,258 (89.3) |
| Smoking status |  |  |  |  |  |  |  |  |  |  |
| Never | N (%) | 30,460 (61.6) | 28,028 (59.2) | 15,455 (58.8) | 21,725 (62.0) | 15,985 (57.9) | 8,051 (57.1) | 5,808 (57.6) | 8,956 (56.5) | 9,652 (56.5) |
| Previous | N (%) | 14,529 (29.4) | 15,477 (32.7) | 8,869 (33.8) | 10,330 (29.5) | 9,317 (33.8) | 4,851 (34.4) | 3,106 (30.8) | 5,245 (33.1) | 5,881 (34.4) |
| Current | N (%) | 4,449 (9) | 3,809 (8.1) | 1,955 (7.4) | 2,993 (8.5) | 2,304 (8.4) | 1,205 (8.5) | 1,169 (11.6) | 1,644 (10.4) | 1,553 (9.1) |
| Sleep pattern |  |  |  |  |  |  |  |  |  |  |
| Healthy | N (%) | 18,445 (37.3) | 14,403 (30.4) | 5,760 (21.9) | 11,933 (34.1) | 6,931 (25.1) | 2,545 (18.0) | 3,697 (36.7) | 4,706 (29.7) | 3,662 (21.4) |
| Intermediate | N (%) | 28,028 (56.7) | 28,685 (60.6) | 16,497 (62.8) | 20,648 (58.9) | 17,441 (63.2) | 8,867 (62.9) | 5,744 (57.0) | 9,601 (60.6) | 10,661 (62.4) |
| Poor | N (%) | 2,965 (6.0) | 4,226 (8.9) | 4,022 (15.3) | 2,467 (7.0) | 3,234 (11.7) | 2,695 (19.1) | 642 (6.4) | 1,538 (9.7) | 2,763 (16.2) |
| Diet pattern |  |  |  |  |  |  |  |  |  |  |
| Good | N (%) | 8,399 (17.0) | 7,777 (16.4) | 3,980 (15.2) | 5,458 (15.6) | 4,452 (16.1) | 2,227 (15.8) | 1,787 (17.7) | 2,733 (17.3) | 2,658 (15.6) |
| Reasonable | N (%) | 37,016 (74.9) | 35,376 (74.8) | 19,381 (73.8) | 26,326 (75.1) | 20,512 (74.3) | 10,284 (72.9) | 7,521 (74.6) | 11,693 (73.8) | 12,483 (73.1) |
| Poor | N (%) | 4,023 (8.1) | 4,161 (8.8) | 2,918 (11.1) | 3,264 (9.3) | 2,642 (9.6) | 1,596 (11.3) | 775 (7.7) | 1,419 (9.0) | 1,945 (11.4) |
| Self-rated health |  |  |  |  |  |  |  |  |  |  |
| Excellent | N (%) | 12,794 (25.9) | 8,256 (17.5) | 2,098 (8.0) | 8,383 (23.9) | 3,798 (13.8) | 853 (6.1) | 2,501 (24.8) | 2,667 (16.8) | 1,261 (7.4) |
| Good | N (%) | 30,353 (61.4) | 30,431 (64.3) | 14,855 (56.5) | 21,267 (60.7) | 16,918 (61.3) | 6,904 (48.9) | 6,073 (60.2) | 9,912 (62.6) | 8,999 (52.7) |
| Fair | N (%) | 5,484 (11.1) | 7,561 (16.0) | 7,601 (28.9) | 4,630 (13.2) | 5,848 (21.2) | 4,932 (35.0) | 1,292 (12.8) | 2,845 (18.0) | 5,477 (32.1) |
| Poor | N (%) | 807 (1.6) | 1,066 (2.3) | 1,725 (6.6) | 768 (2.2) | 1,042 (3.8) | 1,418 (10.1) | 217 (2.2) | 421 (2.7) | 1,349 (7.9) |
| Employment status |  |  |  |  |  |  |  |  |  |  |
| Employed or self-employed | N (%) | 29,420 (59.5) | 25,177 (53.2) | 13,658 (52.0) | 21,448 (61.2) | 14,266 (51.7) | 6,648 (47.1) | 6,214 (61.6) | 9,251 (58.4) | 9,782 (57.3) |
| Retired | N (%) | 15,774 (31.9) | 18,626 (39.4) | 10,061 (38.3) | 10,394 (29.7) | 11,066 (40.1) | 5,724 (40.6) | 2,977 (29.5) | 5,323 (33.6) | 5,367 (31.4) |
| Unemployed or other | N (%) | 4,244 (8.6) | 3,511 (7.4) | 2,560 (9.7) | 3,206 (9.2) | 2,274 (8.2) | 1,735 (12.3) | 892 (8.9) | 1,271 (8.0) | 1,937 (11.3) |

Supplementary Table 4. Adulthood BMI and body composition in each adulthood weight status and/or comparative body weight at age 10 years group (Males)

|  |  | BMI (kg/m^2^) | Fat mass (kg) | Percent fat mass | Trunk fat mass (kg) | Percent trunk fat mass |
| --- | --- | --- | --- | --- | --- | --- |
|  |  | Median (IQR) | Median (IQR) | Median (IQR) | Median (IQR) | Median (IQR) |
| Weight status in adulthood | Comparative body weight at age 10 years |  |  |  |  |  |
| Normal weight | -- | 23.5 (22.4, 24.3) | 14.3 (11.8, 16.7) | 19.8 (16.7, 22.5) | 9.0 (7.2, 10.8) | 21.7 (17.8, 25.2) |
| Overweight | -- | 27.2 (26.1, 28.5) | 21.1 (18.5, 23.9) | 25.2 (22.6, 27.6) | 13.3 (11.5, 15.1) | 27.7 (24.7, 30.6) |
| Obesity | -- | 32.3 (30.9, 34.6) | 30.7 (27.3, 35.4) | 30.9 (28.4, 33.6) | 19.0 (16.9, 21.7) | 34.0 (31.2, 36.8) |
| -- | About average weight | 27.4 (25.2, 29.9) | 21.0 (16.7, 26.1) | 25.1 (21.4, 28.7) | 13.3 (10.5, 16.4) | 27.7 (23.3, 31.7) |
| -- | Thinner | 26.4 (24.3, 29.0) | 20.3 (16.0, 25.3) | 24.8 (21.1, 28.5) | 12.9 (10.1, 15.9) | 27.4 (23.1, 31.5) |
| -- | Plumper | 29.4 (26.8, 32.7) | 25.2 (20.0, 31.6) | 27.8 (24.0, 31.6) | 15.8 (12.6, 19.6) | 30.7 (26.5, 34.7) |
| Normal weight | About average weight | 23.6 (22.6, 24.4) | 14.0 (11.6, 16.3) | 19.4 (16.4, 22.1) | 8.8 (7.0, 10.5) | 21.2 (17.3, 24.7) |
| Normal weight | Thinner | 23.4 (22.2, 24.2) | 14.6 (12.0, 17.0) | 20.1 (17.1, 22.9) | 9.3 (7.4, 11.0) | 22.2 (18.3, 25.6) |
| Normal weight | Plumper | 23.7 (22.7, 24.4) | 14.7 (12.0, 17.1) | 20.0 (16.9, 22.7) | 9.3 (7.4, 11.1) | 22.1 (18.1, 25.5) |
| Overweight | About average weight | 27.3 (26.2, 28.5) | 20.8 (18.2, 23.5) | 24.8 (22.3, 27.3) | 13.1 (11.3, 14.9) | 27.4 (24.3, 30.2) |
| Overweight | Thinner | 27.1 (26.0, 28.3) | 21.5 (18.8, 24.2) | 25.5 (23.1, 28.0) | 13.6 (11.8, 15.3) | 28.1 (25.2, 30.9) |
| Overweight | Plumper | 27.7 (26.5, 28.8) | 21.8 (19.1, 24.6) | 25.6 (23.1, 28.0) | 13.8 (12.0, 15.6) | 28.3 (25.3, 31.1) |
| Obesity | About average weight | 32.1 (30.8, 34.1) | 30.0 (26.8, 34.3) | 30.5 (28.1, 33.1) | 18.6 (16.6, 21.1) | 33.5 (30.8, 36.2) |
| Obesity | Thinner | 32.1 (30.9, 34.3) | 30.8 (27.5, 35.3) | 31.2 (28.8, 33.8) | 19.1 (17.0, 21.6) | 34.2 (31.5, 36.9) |
| Obesity | Plumper | 33.1 (31.3, 35.9) | 32.3 (28.1, 38.0) | 31.7 (28.9, 34.5) | 19.9 (17.5, 23.1) | 34.8 (31.8, 37.7) |

Supplementary Table 5. Adulthood BMI and body composition in each adulthood weight status and/or comparative body weight at age 10 years group (Females)

|  |  | BMI (kg/m^2^) | Fat mass (kg) | Percent fat mass | Trunk fat mass (kg) | Percent trunk fat mass |
| --- | --- | --- | --- | --- | --- | --- |
|  |  | Median (IQR) | Median (IQR) | Median (IQR) | Median (IQR) | Median (IQR) |
| Weight status in adulthood | Comparative body weight at age 10 years |  |  |  |  |  |
| Normal weight | -- | 22.9 (21.6, 23.9) | 18.9 (16.0, 21.6) | 31.1 (27.8, 34.1) | 9.5 (7.6, 11.3) | 28.4 (24.3, 32.2) |
| Overweight | -- | 27.0 (25.9, 28.3) | 27.0 (24.2, 30.0) | 38.0 (35.6, 40.3) | 13.9 (12.1, 15.9) | 35.9 (32.6, 39.0) |
| Obesity | -- | 33.1 (31.3, 36.2) | 38.6 (34.3, 44.4) | 44.4 (41.9, 47.0) | 19.6 (17.0, 22.6) | 42.1 (38.9, 45.3) |
| -- | About average weight | 25.9 (23.5, 29.3) | 24.8 (19.6, 31.3) | 36.3 (31.6, 40.8) | 12.7 (9.7, 16.2) | 33.9 (28.6, 38.9) |
| -- | Thinner | 25.4 (22.9, 28.6) | 24.5 (19.5, 30.8) | 36.4 (31.8, 40.8) | 12.6 (9.7, 16.1) | 34.2 (29.0, 39.1) |
| -- | Plumper | 28.4 (25.2, 32.7) | 29.3 (22.9, 37.6) | 39.3 (34.5, 43.9) | 15.0 (11.5, 19.2) | 37.1 (31.7, 42.0) |
| Normal weight | About average weight | 22.9 (21.7, 24.0) | 18.7 (15.8, 21.4) | 30.8 (27.5, 33.7) | 9.3 (7.5, 11.1) | 28.0 (23.9, 31.7) |
| Normal weight | Thinner | 22.7 (21.3, 23.8) | 19.1 (16.1, 21.9) | 31.6 (28.2, 34.5) | 9.7 (7.8, 11.5) | 29.1 (24.9, 32.8) |
| Normal weight | Plumper | 23.2 (21.9, 24.1) | 19.1 (16.2, 21.7) | 31.2 (28.0, 34.1) | 9.5 (7.7, 11.3) | 28.5 (24.5, 32.3) |
| Overweight | About average weight | 27.0 (25.9, 28.3) | 26.8 (24.0, 29.7) | 37.7 (35.3, 40.0) | 13.7 (11.9, 15.7) | 35.5 (32.2, 38.6) |
| Overweight | Thinner | 27.0 (25.9, 28.3) | 27.4 (24.6, 30.4) | 38.5 (36.2, 40.7) | 14.2 (12.3, 16.2) | 36.5 (33.4, 39.5) |
| Overweight | Plumper | 27.3 (26.1, 28.6) | 27.2 (24.4, 30.3) | 38.0 (35.5, 40.4) | 14.0 (12.1, 16.0) | 35.8 (32.5, 39.0) |
| Obesity | About average weight | 32.8 (31.2, 35.6) | 37.9 (33.9, 43.3) | 44.0 (41.6, 46.5) | 19.2 (16.8, 22.1) | 41.7 (38.6, 44.8) |
| Obesity | Thinner | 32.8 (31.2, 35.6) | 38.3 (34.3, 43.6) | 44.5 (42.2, 47.0) | 19.6 (17.1, 22.5) | 42.4 (39.3, 45.5) |
| Obesity | Plumper | 34.0 (31.7, 37.6) | 40.1 (35.1, 46.9) | 44.8 (42.2, 47.7) | 20.2 (17.4, 23.5) | 42.5 (39.2, 45.7) |

Supplementary Table 6. Numbers of participants, deaths, and events in each adulthood weight status and/or comparative body weight at age 10 years group

|  |  | All-cause mortality | | | | CVD | | | | Obesity-related cancer | | | |
| --- | --- | --- | --- | --- | --- | --- | --- | --- | --- | --- | --- | --- | --- |
|  |  | Males | | Females | | Males | | Females | | Males | | Females | |
| Weight status in adulthood | Comparative body weight  at age 10 years | N | N (%) deaths | N | N (%) deaths | N | N (%) events | N | N (%) events | N | N (%) events | N | N (%) events |
| Normal weight | -- | 46,192 | 4,005 (8.7) | 94,290 | 4,634 (4.9) | 43,941 | 13,508 (30.7) | 91,181 | 20,256 (22.2) | 46,347 | 860 (1.9) | 94,283 | 1,848 (2.0) |
| Overweight | -- | 95,337 | 8,705 (9.1) | 90,502 | 5,415 (6.0) | 89,711 | 32,392 (36.1) | 86,575 | 25,266 (29.2) | 95,547 | 2,355 (2.5) | 90,442 | 2,223 (2.5) |
| Obesity | -- | 48,465 | 6,120 (12.6) | 57,275 | 4,556 (8.0) | 44,635 | 20,127 (45.1) | 53,734 | 19,825 (36.9) | 48,655 | 1,588 (3.3) | 57,217 | 1,886 (3.3) |
| -- | About average | 97,618 | 9,685 (9.9) | 122,658 | 7,045 (5.7) | 91,765 | 33,760 (36.8) | 117,630 | 31,979 (27.2) | 97,931 | 2,511 (2.6) | 122,594 | 2,969 (2.4) |
| -- | Thinner | 66,392 | 6,425 (9.7) | 76,533 | 4,710 (6.2) | 62,237 | 22,985 (36.9) | 72,978 | 21,496 (29.5) | 66,555 | 1,578 (2.4) | 76,497 | 1,847 (2.4) |
| -- | Plumper | 25,984 | 2,720 (10.5) | 42,876 | 2,850 (6.6) | 24,285 | 9,282 (38.2) | 40,882 | 11,872 (29.0) | 26,063 | 714 (2.7) | 42,851 | 1,141 (2.7) |
| Normal weight | About average | 21,500 | 2,005 (9.3) | 49,291 | 2,392 (4.9) | 20,475 | 6,357 (31.0) | 47,733 | 10,457 (21.9) | 21,582 | 414 (1.9) | 49,295 | 960 (2.0) |
| Normal weight | Thinner | 21,749 | 1,754 (8.1) | 34,948 | 1,667 (4.8) | 20,672 | 6,334 (30.6) | 33,743 | 7,685 (22.8) | 21,817 | 393 (1.8) | 34,938 | 674 (1.9) |
| Normal weight | Plumper | 2,943 | 246 (8.4) | 10,051 | 575 (5.7) | 2,794 | 817 (29.2) | 9,705 | 2,114 (21.8) | 2,948 | 53 (1.8) | 10,050 | 214 (2.1) |
| Overweight | About average | 51,897 | 4,770 (9.2) | 47,183 | 2,711 (5.7) | 48,891 | 17,452 (35.7) | 45,262 | 12,756 (28.2) | 52,026 | 1,293 (2.5) | 47,138 | 1,174 (2.5) |
| Overweight | Thinner | 32,244 | 2,912 (9.0) | 27,525 | 1,753 (6.4) | 30,261 | 11,307 (37.4) | 26,172 | 8,421 (32.2) | 32,302 | 785 (2.4) | 27,516 | 692 (2.5) |
| Overweight | Plumper | 11,196 | 1,023 (9.1) | 15,794 | 951 (6.0) | 10,559 | 3,633 (34.4) | 15,141 | 4,089 (27.0) | 11,219 | 277 (2.5) | 15,788 | 357 (2.3) |
| Obesity | About average | 24,221 | 2,910 (12) | 26,184 | 1,942 (7.4) | 22,399 | 9,951 (44.4) | 24,635 | 8,766 (35.6) | 24,323 | 804 (3.3) | 26,161 | 835 (3.2) |
| Obesity | Thinner | 12,399 | 1,759 (14.2) | 14,060 | 1,290 (9.2) | 11,304 | 5,344 (47.3) | 13,063 | 5,390 (41.3) | 12,436 | 400 (3.2) | 14,043 | 481 (3.4) |
| Obesity | Plumper | 11,845 | 1,451 (12.2) | 17,031 | 1,324 (7.8) | 10,932 | 4,832 (44.2) | 16,036 | 5,669 (35.4) | 11,896 | 384 (3.2) | 17,013 | 570 (3.4) |

Supplementary Table 7. Associations of adulthood overweight and obesity with all-cause mortality according to, and testing for effect modification by, comparative body weight at age 10 years (adjusted for adulthood variables)

|  | Males | | | | | |
| --- | --- | --- | --- | --- | --- | --- |
|  | Stratum-specific estimate | | Interaction | | RERI | |
|  | HR (95% CI) | P-value | HR (95% CI) | P-value | Estimate (95% CI) | P-value |
| Overweight |  |  |  |  |  |  |
| If about average weight | 0.95 (0.90, 1.00) | 0.069 | -- |  | -- |  |
| If thinner | 0.97 (0.91, 1.03) | 0.290 | 1.02 (0.94, 1.10) | 0.683 | 0.01 (-0.06, 0.09) | 0.783 |
| If plumper | 0.97 (0.85, 1.12) | 0.708 | 1.02 (0.88, 1.19) | 0.772 | 0.01 (-0.13, 0.16) | 0.865 |
| Obesity |  |  |  |  |  |  |
| If about average weight | 1.08 (1.02, 1.14) | 0.012 | -- |  | -- |  |
| If thinner | 1.21 (1.13, 1.29) | <0.001 | 1.12 (1.03, 1.22) | 0.010 | 0.11 (0.03, 0.20) | 0.012 |
| If plumper | 1.18 (1.03, 1.35) | 0.016 | 1.10 (0.95, 1.27) | 0.212 | 0.10 (-0.05, 0.25) | 0.175 |
| LRT vs model without interactions (p=0.052) |  |  |  |  |  |  |

|  | Females | | | | | |
| --- | --- | --- | --- | --- | --- | --- |
|  | Stratum-specific estimate | | Interaction | | RERI |  |
|  | HR (95% CI) | P-value | HR (95% CI) | P-value | Estimate (95% CI) | P-value |
| Overweight |  |  |  |  |  |  |
| If about average weight | 0.99 (0.94, 1.05) | 0.691 | -- |  | -- |  |
| If thinner | 0.99 (0.93, 1.06) | 0.850 | 1.00 (0.92, 1.10) | 0.916 | 0.00 (-0.09, 0.09) | 0.967 |
| If plumper | 0.92 (0.83, 1.03) | 0.137 | 0.93 (0.83, 1.05) | 0.259 | -0.08 (-0.21, 0.05) | 0.241 |
| Obesity |  |  |  |  |  |  |
| If about average weight | 1.12 (1.05, 1.19) | <0.001 | -- |  | -- |  |
| If thinner | 1.18 (1.09, 1.27) | <0.001 | 1.05 (0.96, 1.16) | 0.267 | 0.06 (-0.04, 0.17) | 0.224 |
| If plumper | 1.08 (0.98, 1.19) | 0.135 | 0.97 (0.86, 1.08) | 0.555 | -0.02 (-0.16, 0.11) | 0.761 |
| LRT vs model without interactions (p=0.551) |  |  |  |  |  |  |

LRT, likelihood-ratio test; RERI, relative excess risk due to interaction

Referent: normal weight in adulthood.

Models adjusted for age, ethnicity, relative age voice break (males) or age at menarche (females), comparative height at age 10 years, alcohol status, smoking status, sleep pattern, diet pattern, self-rated health, employment status, and Townsend index quintile.

Supplementary Table 8. Associations of adulthood overweight and obesity with all-cause mortality according to, and testing for effect modification by, comparative body weight at age 10 years (adjusted for adulthood variables including MET minutes per week for moderate and vigorous physical activity)

|  | Males | | | | | |
| --- | --- | --- | --- | --- | --- | --- |
|  | Stratum-specific estimate | | Interaction | | RERI | |
|  | HR (95% CI) | P-value | HR (95% CI) | P-value | Estimate (95% CI) | P-value |
| Overweight |  |  |  |  |  |  |
| If about average weight | 0.97 (0.91, 1.02) | 0.242 | -- |  | -- |  |
| If thinner | 0.99 (0.92, 1.05) | 0.684 | 1.02 (0.94, 1.11) | 0.636 | 0.02 (-0.07, 0.10) | 0.725 |
| If plumper | 0.98 (0.84, 1.14) | 0.779 | 1.01 (0.86, 1.19) | 0.875 | 0.00 (-0.16, 0.17) | 0.969 |
| Obesity |  |  |  |  |  |  |
| If about average weight | 1.08 (1.01, 1.15) | 0.017 | -- |  | -- |  |
| If thinner | 1.24 (1.15, 1.34) | <0.001 | 1.14 (1.04, 1.26) | 0.007 | 0.14 (0.04, 0.24) | 0.006 |
| If plumper | 1.15 (0.99, 1.33) | 0.064 | 1.06 (0.91, 1.25) | 0.456 | 0.07 (-0.10, 0.24) | 0.404 |
| LRT vs model without interactions (p=0.055) |  |  |  |  |  |  |

|  | Females | | | | | |
| --- | --- | --- | --- | --- | --- | --- |
|  | Stratum-specific estimate | | Interaction | | RERI |  |
|  | HR (95% CI) | P-value | HR (95% CI) | P-value | Estimate (95% CI) | P-value |
| Overweight |  |  |  |  |  |  |
| If about average weight | 0.99 (0.93, 1.06) | 0.839 | -- |  | -- |  |
| If thinner | 0.98 (0.91, 1.07) | 0.688 | 0.99 (0.89, 1.10) | 0.853 | -0.01 (-0.11, 0.09) | 0.829 |
| If plumper | 0.89 (0.78, 1.00) | 0.053 | 0.89 (0.78, 1.02) | 0.106 | -0.13 (-0.28, 0.03) | 0.108 |
| Obesity |  |  |  |  |  |  |
| If about average weight | 1.11 (1.03, 1.19) | 0.007 | -- |  | -- |  |
| If thinner | 1.18 (1.08, 1.29) | <0.001 | 1.06 (0.95, 1.19) | 0.292 | 0.07 (-0.05, 0.19) | 0.272 |
| If plumper | 1.04 (0.93, 1.17) | 0.477 | 0.94 (0.82, 1.08) | 0.385 | -0.05 (-0.21, 0.11) | 0.509 |
| LRT vs model without interactions (p=0.348) |  |  |  |  |  |  |

LRT, likelihood-ratio test; RERI, relative excess risk due to interaction

Referent: normal weight in adulthood.

Models adjusted for age, ethnicity, relative age voice break (males) or age at menarche (females), comparative height at age 10 years, alcohol status, smoking status, sleep pattern, diet pattern, self-rated health, employment status, Townsend index quintile, and MET minutes per week for moderate and vigorous physical activity.

Supplementary Table 9. Models for all-cause mortality examining the interaction between adult BMI and comparative body weight at age 10 years

|  | Males | | Females | |
| --- | --- | --- | --- | --- |
|  | HR (95% CI) | P-value | HR (95% CI) | P-value |
| Adult BMI | 1.04 (1.03, 1.04) | <0.001 | 1.03 (1.03, 1.04) | <0.001 |
| Comparative child body weight |  |  |  |  |
| About average weight (referent) | -- |  | -- |  |
| Thinner | 1.02 (0.99, 1.06) | 0.195 | 1.09 (1.05, 1.13) | <0.001 |
| Plumper | 1.05 (0.99, 1.10) | 0.092 | 1.16 (1.10, 1.21) | <0.001 |
| Interactions |  |  |  |  |
| Adult BMI*Thinner | 1.02 (1.01, 1.02) | <0.001 | 1.01 (1.00, 1.02) | 0.022 |
| Adult BMI*Plumper | 1.01 (1.00, 1.02) | 0.035 | 1.00 (1.00, 1.01) | 0.410 |

Models adjusted for age, ethnicity, relative age voice break (males) or age at menarche (females), and comparative height at age 10 years.

Supplementary Table 10. Associations of adulthood overweight and obesity with all-cause mortality according to, and testing for effect modification by, comparative body weight at age 10 years (defined using the child polygenic risk score)

|  | Males | | | | | |
| --- | --- | --- | --- | --- | --- | --- |
|  | Stratum-specific estimate | | Interaction | | RERI | |
|  | HR (95% CI) | P-value | HR (95% CI) | P-value | Estimate (95% CI) | P-value |
| Overweight |  |  |  |  |  |  |
| If average (using child PRS) | 1.01 (0.96, 1.07) | 0.605 | -- |  | -- |  |
| If thinner (using child PRS) | 0.93 (0.87, 0.99) | 0.021 | 0.92 (0.84, 0.99) | 0.036 | -0.09 (-0.17, 0.00) | 0.043 |
| If plumper (using child PRS) | 1.08 (0.97, 1.20) | 0.157 | 1.06 (0.95, 1.20) | 0.302 | 0.07 (-0.05, 0.19) | 0.277 |
| Obesity |  |  |  |  |  |  |
| If average (using child PRS) | 1.44 (1.36, 1.53) | <0.001 | -- |  | -- |  |
| If thinner (using child PRS) | 1.38 (1.29, 1.48) | <0.001 | 0.96 (0.88, 1.05) | 0.353 | -0.05 (-0.16, 0.06) | 0.358 |
| If plumper (using child PRS) | 1.45 (1.30, 1.62) | <0.001 | 1.00 (0.89, 1.14) | 0.949 | 0.02 (-0.13, 0.17) | 0.813 |
| LRT vs model without interactions (p=0.066) |  |  |  |  |  |  |

|  | Females | | | | | |
| --- | --- | --- | --- | --- | --- | --- |
|  | Stratum-specific estimate | | Interaction | | RERI |  |
|  | HR (95% CI) | P-value | HR (95% CI) | P-value | Estimate (95% CI) | P-value |
| Overweight |  |  |  |  |  |  |
| If average (using child PRS) | 1.05 (0.99, 1.11) | 0.121 | -- |  | -- |  |
| If thinner (using child PRS) | 1.07 (1.00, 1.15) | 0.066 | 1.02 (0.93, 1.12) | 0.655 | 0.02 (-0.07, 0.11) | 0.649 |
| If plumper (using child PRS) | 1.05 (0.96, 1.16) | 0.280 | 1.01 (0.90, 1.12) | 0.905 | 0.01 (-0.11, 0.13) | 0.852 |
| Obesity |  |  |  |  |  |  |
| If average (using child PRS) | 1.51 (1.42, 1.60) | <0.001 | -- |  | -- |  |
| If thinner (using child PRS) | 1.45 (1.34, 1.57) | <0.001 | 0.96 (0.88, 1.06) | 0.454 | -0.05 (-0.18, 0.07) | 0.392 |
| If plumper (using child PRS) | 1.40 (1.27, 1.54) | <0.001 | 0.93 (0.83, 1.04) | 0.200 | -0.07 (-0.22, 0.07) | 0.319 |
| LRT vs model without interactions (p=0.529) |  |  |  |  |  |  |

LRT, likelihood-ratio test; PRS, polygenic risk score; RERI, relative excess risk due to interaction

Referent: normal weight in adulthood.

Models adjusted for age, ethnicity, relative age voice break (males) or age at menarche (females), and comparative height at age 10 years.

Supplementary Table 11. Models for all all-cause mortality examining 1) the interaction between child and adult polygenic risk scores and 2) interactions between adulthood weight status and the child polygenic risk score

|  | Males | | Females | |
| --- | --- | --- | --- | --- |
|  | HR (95% CI) | P-value | HR (95% CI) | P-value |
| Child PRS (mean 0, SD 1) | 1.02 (1.00, 1.03) | 0.035 | 1.01 (1.00, 1.03) | 0.114 |
| Adult PRS (mean 0, SD 1) | 1.06 (1.04, 1.07) | <0.001 | 1.06 (1.04, 1.07) | <0.001 |
| Interaction |  |  |  |  |
| Child PRS*Adult PRS | 1.00 (0.99, 1.01) | 0.904 | 1.01 (1.00, 1.03) | 0.115 |
| Adult weight status |  |  |  |  |
| Normal weight (referent) | -- |  | -- |  |
| Overweight | 0.99 (0.96, 1.03) | 0.683 | 1.05 (1.01, 1.10) | 0.010 |
| Obesity | 1.42 (1.36, 1.48) | <0.001 | 1.47 (1.40, 1.53) | <0.001 |
| Child PRS (mean 0, SD 1) | 1.00 (0.97, 1.03) | 0.977 | 1.03 (1.00, 1.06) | 0.035 |
| Interactions |  |  |  |  |
| Overweight*Child PRS | 1.04 (1.00, 1.08) | 0.028 | 0.98 (0.95, 1.02) | 0.422 |
| Obesity*Child PRS | 1.02 (0.98, 1.06) | 0.305 | 1.00 (0.96, 1.04) | 0.872 |

PRS, polygenic risk score

Models adjusted for age, ethnicity, relative age voice break (males) or age at menarche (females), and comparative height at age 10 years.

Supplementary Table 12. Associations of adulthood overweight and obesity with all-cause mortality according to, and testing for effect modification by, comparative body weight at age 10 years: observational and genetic analyses in participants with White ethnicity

|  | Males | | | | | |
| --- | --- | --- | --- | --- | --- | --- |
|  | Stratum-specific estimate | | Interaction | | RERI | |
|  | HR (95% CI) | P-value | HR (95% CI) | P-value | Estimate (95% CI) | P-value |
| Overweight |  |  |  |  |  |  |
| If average | 0.94 (0.89, 0.99) | 0.026 | -- |  | -- |  |
| If thinner | 1.02 (0.96, 1.09) | 0.442 | 1.09 (1.00, 1.18) | 0.040 | 0.08 (0.01, 0.15) | 0.036 |
| If plumper | 1.01 (0.87, 1.16) | 0.918 | 1.07 (0.92, 1.24) | 0.381 | 0.06 (-0.09, 0.22) | 0.406 |
| Obesity |  |  |  |  |  |  |
| If average | 1.28 (1.20, 1.35) | <0.001 | -- |  | -- |  |
| If thinner | 1.64 (1.53, 1.75) | <0.001 | 1.28 (1.17, 1.40) | <0.001 | 0.31 (0.21, 0.41) | <0.001 |
| If plumper | 1.45 (1.26, 1.66) | <0.001 | 1.13 (0.98, 1.32) | 0.096 | 0.18 (0.02, 0.34) | 0.031 |
| LRT vs model without interactions (p<0.001) |  |  |  |  |  |  |
| Overweight (using adult PRS) |  |  |  |  |  |  |
| If average (using child PRS) | 1.06 (1.00, 1.11) | 0.053 | -- |  | -- |  |
| If thinner (using child PRS) | 1.06 (1.01, 1.13) | 0.030 | 1.01 (0.93, 1.09) | 0.841 | 0.01 (-0.07, 0.09) | 0.863 |
| If plumper (using child PRS) | 1.06 (0.93, 1.22) | 0.362 | 1.01 (0.87, 1.17) | 0.904 | 0.01 (-0.14, 0.17) | 0.876 |
| Obesity (using adult PRS) |  |  |  |  |  |  |
| If average (using child PRS) | 1.16 (1.09, 1.23) | <0.001 | -- |  | -- |  |
| If thinner (using child PRS) | 1.12 (1.04, 1.21) | 0.003 | 0.97 (0.88, 1.07) | 0.534 | -0.04 (-0.14, 0.07) | 0.465 |
| If plumper (using child PRS) | 1.16 (1.01, 1.33) | 0.034 | 1.00 (0.86, 1.16) | 0.995 | 0.01 (-0.15, 0.17) | 0.931 |
| LRT vs model without interactions (p=0.939) |  |  |  |  |  |  |

|  | Females | | | | | |
| --- | --- | --- | --- | --- | --- | --- |
|  | Stratum-specific estimate | | Interaction | | RERI |  |
|  | HR (95% CI) | P-value | HR (95% CI) | P-value | Estimate (95% CI) | P-value |
| Overweight |  |  |  |  |  |  |
| If average | 1.04 (0.98, 1.10) | 0.211 | -- |  | -- |  |
| If thinner | 1.10 (1.03, 1.18) | 0.004 | 1.07 (0.98, 1.16) | 0.155 | 0.07 (-0.02, 0.16) | 0.136 |
| If plumper | 0.94 (0.85, 1.04) | 0.247 | 0.91 (0.81, 1.02) | 0.107 | -0.11 (-0.26, 0.03) | 0.123 |
| Obesity |  |  |  |  |  |  |
| If average | 1.39 (1.31, 1.47) | <0.001 | -- |  | -- |  |
| If thinner | 1.60 (1.49, 1.73) | <0.001 | 1.15 (1.05, 1.27) | 0.003 | 0.23 (0.10, 0.35) | <0.001 |
| If plumper | 1.29 (1.17, 1.42) | <0.001 | 0.93 (0.83, 1.04) | 0.211 | -0.02 (-0.18, 0.14) | 0.800 |
| LRT vs model without interactions (p=0.005) |  |  |  |  |  |  |
| Overweight (using adult PRS) |  |  |  |  |  |  |
| If average (using child PRS) | 1.05 (0.99, 1.11) | 0.089 | -- |  | -- |  |
| If thinner (using child PRS) | 1.08 (1.01, 1.16) | 0.020 | 1.03 (0.95, 1.13) | 0.476 | 0.03 (-0.06, 0.12) | 0.455 |
| If plumper (using child PRS) | 0.98 (0.88, 1.09) | 0.739 | 0.94 (0.83, 1.05) | 0.280 | -0.07 (-0.20, 0.06) | 0.296 |
| Obesity (using adult PRS) |  |  |  |  |  |  |
| If average (using child PRS) | 1.17 (1.10, 1.24) | <0.001 | -- |  | -- |  |
| If thinner (using child PRS) | 1.11 (1.01, 1.22) | 0.027 | 0.95 (0.85, 1.06) | 0.339 | -0.06 (-0.18, 0.06) | 0.329 |
| If plumper (using child PRS) | 1.14 (1.03, 1.26) | 0.015 | 0.97 (0.86, 1.10) | 0.640 | -0.02 (-0.16, 0.11) | 0.756 |
| LRT vs model without interactions (p=0.355) |  |  |  |  |  |  |

LRT, likelihood-ratio test; PRS, polygenic risk score; RERI, relative excess risk due to interaction

Referent: normal weight in adulthood.

Models adjusted for age, ethnicity, relative age voice break (males) or age at menarche (females), and comparative height at age 10 years.

Supplementary Table 13. Associations of adulthood overweight and obesity with incident cardiovascular disease according to, and testing for effect modification by, comparative body weight at age 10 years (adjusted for adulthood variables)

|  | Males | | | | | |
| --- | --- | --- | --- | --- | --- | --- |
|  | Stratum-specific estimate | | Interaction | | RERI | |
|  | HR (95% CI) | P-value | HR (95% CI) | P-value | Estimate (95% CI) | P-value |
| Overweight |  |  |  |  |  |  |
| If about average weight | 1.15 (1.12, 1.18) | <0.001 | -- |  | -- |  |
| If thinner | 1.16 (1.13, 1.20) | <0.001 | 1.01 (0.97, 1.06) | 0.515 | 0.02 (-0.03, 0.06) | 0.395 |
| If plumper | 1.14 (1.06, 1.23) | 0.001 | 0.99 (0.91, 1.08) | 0.845 | -0.01 (-0.10, 0.07) | 0.740 |
| Obesity |  |  |  |  |  |  |
| If about average weight | 1.40 (1.36, 1.45) | <0.001 | -- |  | -- |  |
| If thinner | 1.41 (1.36, 1.47) | <0.001 | 1.01 (0.96, 1.06) | 0.738 | 0.02 (-0.04, 0.08) | 0.444 |
| If plumper | 1.48 (1.37, 1.59) | <0.001 | 1.06 (0.97, 1.14) | 0.185 | 0.07 (-0.02, 0.16) | 0.112 |
| LRT vs model without interactions (p=0.095) |  |  |  |  |  |  |

|  | Females | | | | | |
| --- | --- | --- | --- | --- | --- | --- |
|  | Stratum-specific estimate | | Interaction | | RERI |  |
|  | HR (95% CI) | P-value | HR (95% CI) | P-value | Estimate (95% CI) | P-value |
| Overweight |  |  |  |  |  |  |
| If about average weight | 1.17 (1.14, 1.21) | <0.001 | -- |  | -- |  |
| If thinner | 1.23 (1.19, 1.26) | <0.001 | 1.04 (1.00, 1.09) | 0.038 | 0.06 (0.01, 0.11) | 0.010 |
| If plumper | 1.17 (1.11, 1.23) | <0.001 | 0.99 (0.94, 1.05) | 0.845 | -0.01 (-0.07, 0.06) | 0.862 |
| Obesity |  |  |  |  |  |  |
| If about average weight | 1.41 (1.37, 1.46) | <0.001 | -- |  | -- |  |
| If thinner | 1.47 (1.42, 1.53) | <0.001 | 1.04 (1.00, 1.09) | 0.071 | 0.09 (0.03, 0.15) | 0.003 |
| If plumper | 1.49 (1.42, 1.57) | <0.001 | 1.05 (0.99, 1.12) | 0.078 | 0.07 (0.01, 0.14) | 0.035 |
| LRT vs model without interactions (p=0.021) |  |  |  |  |  |  |

LRT, likelihood-ratio test; RERI, relative excess risk due to interaction

Referent: normal weight in adulthood.

Models adjusted for age, ethnicity, relative age voice break (males) or age at menarche (females), comparative height at age 10 years, alcohol status, smoking status, sleep pattern, diet pattern, self-rated health, employment status, and Townsend index quintile.

Supplementary Table 14. Associations of adulthood overweight and obesity with incident cardiovascular disease according to, and testing for effect modification by, comparative body weight at age 10 years (adjusted for adulthood variables including MET minutes per week for moderate and vigorous physical activity)

|  | Males | | | | | |
| --- | --- | --- | --- | --- | --- | --- |
|  | Stratum-specific estimate | | Interaction | | RERI | |
|  | HR (95% CI) | P-value | HR (95% CI) | P-value | Estimate (95% CI) | P-value |
| Overweight |  |  |  |  |  |  |
| If about average weight | 1.15 (1.11, 1.19) | <0.001 | -- |  | -- |  |
| If thinner | 1.18 (1.14, 1.22) | <0.001 | 1.02 (0.98, 1.07) | 0.353 | 0.03 (-0.02, 0.08) | 0.246 |
| If plumper | 1.15 (1.06, 1.25) | 0.001 | 1.00 (0.92, 1.09) | 0.995 | -0.01 (-0.10, 0.08) | 0.900 |
| Obesity |  |  |  |  |  |  |
| If about average weight | 1.41 (1.36, 1.46) | <0.001 | -- |  | -- |  |
| If thinner | 1.40 (1.35, 1.46) | <0.001 | 1.00 (0.95, 1.05) | 0.938 | 0.01 (-0.06, 0.08) | 0.774 |
| If plumper | 1.49 (1.37, 1.61) | <0.001 | 1.06 (0.97, 1.15) | 0.206 | 0.07 (-0.02, 0.16) | 0.143 |
| LRT vs model without interactions (p=0.094) |  |  |  |  |  |  |

|  | Females | | | | | |
| --- | --- | --- | --- | --- | --- | --- |
|  | Stratum-specific estimate | | Interaction | | RERI |  |
|  | HR (95% CI) | P-value | HR (95% CI) | P-value | Estimate (95% CI) | P-value |
| Overweight |  |  |  |  |  |  |
| If about average weight | 1.19 (1.15, 1.22) | <0.001 | -- |  | -- |  |
| If thinner | 1.23 (1.19, 1.28) | <0.001 | 1.04 (0.99, 1.09) | 0.098 | 0.06 (0.01, 0.11) | 0.029 |
| If plumper | 1.15 (1.08, 1.22) | <0.001 | 0.97 (0.91, 1.04) | 0.391 | -0.03 (-0.10, 0.04) | 0.404 |
| Obesity |  |  |  |  |  |  |
| If about average weight | 1.43 (1.39, 1.49) | <0.001 | -- |  | -- |  |
| If thinner | 1.47 (1.41, 1.53) | <0.001 | 1.02 (0.97, 1.08) | 0.409 | 0.06 (-0.01, 0.14) | 0.081 |
| If plumper | 1.19 (1.15, 1.22) | <0.001 | 1.02 (0.95, 1.09) | 0.569 | 0.03 (-0.05, 0.11) | 0.470 |
| LRT vs model without interactions (p=0.128) |  |  |  |  |  |  |

LRT, likelihood-ratio test; RERI, relative excess risk due to interaction

Referent: normal weight in adulthood.

Models adjusted for age, ethnicity, relative age voice break (males) or age at menarche (females), comparative height at age 10 years, alcohol status, smoking status, sleep pattern, diet pattern, self-rated health, employment status, Townsend index quintile, and MET minutes per week for moderate and vigorous physical activity.

Supplementary Table 15. Models for incident cardiovascular disease examining the interaction between adult BMI and comparative body weight at age 10 years

|  | Males | | Females | |
| --- | --- | --- | --- | --- |
|  | HR (95% CI) | P-value | HR (95% CI) | P-value |
| Adult BMI | 1.05 (1.04, 1.05) | <0.001 | 1.04 (1.04, 1.05) | <0.001 |
| Comparative child body weight |  |  |  |  |
| About average weight (referent) | -- |  | -- |  |
| Thinner | 1.07 (1.05, 1.08) | <0.001 | 1.13 (1.11, 1.15) | <0.001 |
| Plumper | 1.00 (0.98, 1.03) | 0.795 | 1.01 (0.99, 1.04) | 0.261 |
| Interactions |  |  |  |  |
| Adult BMI*Thinner | 1.01 (1.00, 1.01) | 0.013 | 1.01 (1.00, 1.01) | <0.001 |
| Adult BMI*Plumper | 1.00 (1.00, 1.01) | 0.790 | 1.00 (1.00, 1.00) | 0.843 |

Models adjusted for age, ethnicity, relative age voice break (males) or age at menarche (females), and comparative height at age 10 years.

Supplementary Table 16. Associations of adulthood overweight and obesity with incident cardiovascular disease according to, and testing for effect modification by, comparative body weight at age 10 years (defined using the child polygenic risk score)

|  | Males | | | | | |
| --- | --- | --- | --- | --- | --- | --- |
|  | Stratum-specific estimate | | Interaction | | RERI | |
|  | HR (95% CI) | P-value | HR (95% CI) | P-value | Estimate (95% CI) | P-value |
| Overweight |  |  |  |  |  |  |
| If average (using child PRS) | 1.19 (1.16, 1.23) | <0.001 | -- |  | -- |  |
| If thinner (using child PRS) | 1.16 (1.12, 1.20) | <0.001 | 0.97 (0.93, 1.02) | 0.222 | -0.03 (-0.08, 0.02) | 0.246 |
| If plumper (using child PRS) | 1.21 (1.14, 1.28) | <0.001 | 1.01 (0.95, 1.08) | 0.675 | 0.01 (-0.05, 0.08) | 0.684 |
| Obesity |  |  |  |  |  |  |
| If average (using child PRS) | 1.62 (1.57, 1.67) | <0.001 | -- |  | -- |  |
| If thinner (using child PRS) | 1.57 (1.51, 1.63) | <0.001 | 0.97 (0.93, 1.02) | 0.234 | -0.03 (-0.09, 0.03) | 0.354 |
| If plumper (using child PRS) | 1.66 (1.56, 1.76) | <0.001 | 1.03 (0.96, 1.10) | 0.451 | 0.04 (-0.05, 0.12) | 0.408 |
| LRT vs model without interactions (p=0.539) |  |  |  |  |  |  |

|  | Females | | | | | |
| --- | --- | --- | --- | --- | --- | --- |
|  | Stratum-specific estimate | | Interaction | | RERI |  |
|  | HR (95% CI) | P-value | HR (95% CI) | P-value | Estimate (95% CI) | P-value |
| Overweight |  |  |  |  |  |  |
| If average (using child PRS) | 1.26 (1.23, 1.30) | <0.001 | -- |  | -- |  |
| If thinner (using child PRS) | 1.26 (1.22, 1.30) | <0.001 | 1.00 (0.96, 1.04) | 0.898 | 0.00 (-0.05, 0.04) | 0.875 |
| If plumper (using child PRS) | 1.23 (1.18, 1.29) | <0.001 | 0.97 (0.92, 1.03) | 0.314 | -0.03 (-0.09, 0.03) | 0.312 |
| Obesity |  |  |  |  |  |  |
| If average (using child PRS) | 1.74 (1.69, 1.79) | <0.001 | -- |  | -- |  |
| If thinner (using child PRS) | 1.71 (1.65, 1.78) | <0.001 | 0.98 (0.94, 1.03) | 0.503 | -0.03 (-0.09, 0.04) | 0.432 |
| If plumper (using child PRS) | 1.68 (1.60, 1.76) | <0.001 | 0.96 (0.91, 1.02) | 0.173 | -0.06 (-0.13, 0.02) | 0.138 |
| LRT vs model without interactions (p=0.707) |  |  |  |  |  |  |

LRT, likelihood-ratio test; PRS, polygenic risk score; RERI, relative excess risk due to interaction

Referent: normal weight in adulthood.

Models adjusted for age, ethnicity, relative age voice break (males) or age at menarche (females), and comparative height at age 10 years.

Supplementary Table 17. Models for incident cardiovascular disease examining 1) the interaction between child and adult polygenic risk scores and 2) interactions between adulthood weight status and the child polygenic risk score

|  | Males | | Females | |
| --- | --- | --- | --- | --- |
|  | HR (95% CI) | P-value | HR (95% CI) | P-value |
| Child PRS (mean 0, SD 1) | 1.00 (0.99, 1.01) | 0.532 | 1.00 (0.99, 1.01) | 0.585 |
| Adult PRS (mean 0, SD 1) | 1.04 (1.03, 1.04) | <0.001 | 1.03 (1.03, 1.04) | <0.001 |
| Interaction |  |  |  |  |
| Child PRS*Adult PRS | 1.00 (0.99, 1.01) | 0.771 | 1.00 (1.00, 1.01) | 0.329 |
| Adult weight status |  |  |  |  |
| Normal weight (referent) | -- |  | -- |  |
| Overweight | 1.18 (1.16, 1.21) | <0.001 | 1.26 (1.23, 1.28) | <0.001 |
| Obesity | 1.60 (1.57, 1.64) | <0.001 | 1.72 (1.69, 1.76) | <0.001 |
| Child PRS (mean 0, SD 1) | 0.99 (0.97, 1.01) | 0.218 | 1.00 (0.99, 1.01) | 0.940 |
| Interactions |  |  |  |  |
| Overweight*Child PRS | 1.01 (0.99, 1.03) | 0.370 | 0.99 (0.98, 1.01) | 0.546 |
| Obesity*Child PRS | 1.02 (0.99, 1.04) | 0.132 | 1.00 (0.98, 1.02) | 0.640 |

PRS, polygenic risk score

Models adjusted for age, ethnicity, relative age voice break (males) or age at menarche (females), and comparative height at age 10 years.

Supplementary Table 18. Associations of adulthood overweight and obesity with incident cardiovascular disease according to, and testing for effect modification by, comparative body weight at age 10 years: observational and genetic analyses in participants with White ethnicity

|  | Males | | | | | |
| --- | --- | --- | --- | --- | --- | --- |
|  | Stratum-specific estimate | | Interaction | | RERI | |
|  | HR (95% CI) | P-value | HR (95% CI) | P-value | Estimate (95% CI) | P-value |
| Overweight |  |  |  |  |  |  |
| If average | 1.17 (1.14, 1.20) | <0.001 | -- |  | -- |  |
| If thinner | 1.21 (1.17, 1.25) | <0.001 | 1.04 (0.99, 1.08) | 0.109 | 0.05 (0.00, 0.09) | 0.040 |
| If plumper | 1.19 (1.10, 1.29) | <0.001 | 1.02 (0.94, 1.11) | 0.639 | 0.02 (-0.07, 0.10) | 0.667 |
| Obesity |  |  |  |  |  |  |
| If average | 1.56 (1.52, 1.62) | <0.001 | -- |  | -- |  |
| If thinner | 1.66 (1.60, 1.72) | <0.001 | 1.06 (1.01, 1.11) | 0.020 | 0.12 (0.05, 0.18) | 0.001 |
| If plumper | 1.70 (1.58, 1.84) | <0.001 | 1.09 (1.00, 1.18) | 0.043 | 0.12 (0.03, 0.22) | 0.008 |
| LRT vs model without interactions (p=0.020) |  |  |  |  |  |  |
| Overweight (using adult PRS) |  |  |  |  |  |  |
| If average (using child PRS) | 1.03 (1.01, 1.06) | 0.020 | -- |  | -- |  |
| If thinner (using child PRS) | 1.06 (1.03, 1.09) | <0.001 | 1.02 (0.98, 1.07) | 0.248 | 0.02 (-0.02, 0.07) | 0.243 |
| If plumper (using child PRS) | 1.01 (0.94, 1.09) | 0.743 | 0.98 (0.91, 1.06) | 0.574 | -0.02 (-0.10, 0.06) | 0.582 |
| Obesity (using adult PRS) |  |  |  |  |  |  |
| If average (using child PRS) | 1.08 (1.05, 1.12) | <0.001 | -- |  | -- |  |
| If thinner (using child PRS) | 1.11 (1.06, 1.15) | <0.001 | 1.02 (0.97, 1.08) | 0.411 | 0.02 (-0.03, 0.08) | 0.415 |
| If plumper (using child PRS) | 1.06 (0.99, 1.14) | 0.095 | 0.98 (0.91, 1.06) | 0.660 | -0.02 (-0.10, 0.06) | 0.670 |
| LRT vs model without interactions (p=0.698) |  |  |  |  |  |  |

|  | Females | | | | | |
| --- | --- | --- | --- | --- | --- | --- |
|  | Stratum-specific estimate | | Interaction | | RERI |  |
|  | HR (95% CI) | P-value | HR (95% CI) | P-value | Estimate (95% CI) | P-value |
| Overweight |  |  |  |  |  |  |
| If average | 1.23 (1.20, 1.26) | <0.001 | -- |  | -- |  |
| If thinner | 1.32 (1.28, 1.37) | <0.001 | 1.08 (1.04, 1.12) | <0.001 | 0.12 (0.07, 0.17) | <0.001 |
| If plumper | 1.22 (1.16, 1.29) | <0.001 | 1.00 (0.94, 1.06) | 0.923 | 0.00 (-0.06, 0.07) | 0.919 |
| Obesity |  |  |  |  |  |  |
| If average | 1.66 (1.61, 1.71) | <0.001 | -- |  | -- |  |
| If thinner | 1.81 (1.75, 1.88) | <0.001 | 1.09 (1.04, 1.14) | <0.001 | 0.21 (0.14, 0.28) | <0.001 |
| If plumper | 1.76 (1.67, 1.85) | <0.001 | 1.06 (1.00, 1.12) | 0.061 | 0.11 (0.03, 0.18) | 0.006 |
| LRT vs model without interactions (p<0.001) |  |  |  |  |  |  |
| Overweight (using adult PRS) |  |  |  |  |  |  |
| If average (using child PRS) | 1.02 (1.00, 1.05) | 0.097 | -- |  | -- |  |
| If thinner (using child PRS) | 1.03 (1.00, 1.07) | 0.044 | 1.01 (0.97, 1.05) | 0.595 | 0.01 (-0.03, 0.05) | 0.610 |
| If plumper (using child PRS) | 1.00 (0.95, 1.05) | 0.87 | 0.97 (0.92, 1.03) | 0.371 | -0.03 (-0.08, 0.03) | 0.367 |
| Obesity (using adult PRS) |  |  |  |  |  |  |
| If average (using child PRS) | 1.06 (1.03, 1.10) | <0.001 | -- |  | -- |  |
| If thinner (using child PRS) | 1.07 (1.03, 1.12) | 0.002 | 1.01 (0.96, 1.06) | 0.763 | 0.01 (-0.05, 0.06) | 0.804 |
| If plumper (using child PRS) | 1.07 (1.02, 1.13) | 0.006 | 1.01 (0.95, 1.07) | 0.779 | 0.01 (-0.05, 0.07) | 0.777 |
| LRT vs model without interactions (p=0.630) |  |  |  |  |  |  |

LRT, likelihood-ratio test; PRS, polygenic risk score; RERI, relative excess risk due to interaction

Referent: normal weight in adulthood.

Models adjusted for age, ethnicity, relative age voice break (males) or age at menarche (females), and comparative height at age 10 years.

Supplementary Table 19. Associations of adulthood overweight and obesity with incident obesity-related cancer according to, and testing for effect modification by, comparative body weight at age 10 years (adjusted for adulthood variables)

|  | Males | | | | | |
| --- | --- | --- | --- | --- | --- | --- |
|  | Stratum-specific estimate | | Interaction | | RERI | |
|  | HR (95% CI) | P-value | HR (95% CI) | P-value | Estimate (95% CI) | P-value |
| Overweight |  |  |  |  |  |  |
| If about average weight | 1.25 (1.12, 1.40) | <0.001 | -- |  | -- |  |
| If thinner | 1.24 (1.10, 1.40) | 0.001 | 0.99 (0.84, 1.17) | 0.892 | -0.02 (-0.20, 0.16) | 0.828 |
| If plumper | 1.28 (0.95, 1.71) | 0.105 | 1.02 (0.74, 1.39) | 0.914 | 0.02 (-0.31, 0.35) | 0.915 |
| Obesity |  |  |  |  |  |  |
| If about average weight | 1.59 (1.41, 1.80) | <0.001 | -- |  | -- |  |
| If thinner | 1.52 (1.32, 1.75) | <0.001 | 0.95 (0.79, 1.14) | 0.602 | -0.09 (-0.32, 0.14) | 0.457 |
| If plumper | 1.64 (1.23, 2.19) | 0.001 | 1.03 (0.76, 1.41) | 0.850 | 0.05 (-0.30, 0.40) | 0.767 |
| LRT vs model without interactions (p=0.975) |  |  |  |  |  |  |

|  | Females | | | | | |
| --- | --- | --- | --- | --- | --- | --- |
|  | Stratum-specific estimate | | Interaction | | RERI |  |
|  | HR (95% CI) | P-value | HR (95% CI) | P-value | Estimate (95% CI) | P-value |
| Overweight |  |  |  |  |  |  |
| If about average weight | 1.16 (1.06, 1.26) | 0.001 | -- |  | -- |  |
| If thinner | 1.13 (1.01, 1.25) | 0.028 | 0.97 (0.85, 1.11) | 0.694 | -0.03 (-0.18, 0.12) | 0.683 |
| If plumper | 1.00 (0.84, 1.18) | 0.966 | 0.86 (0.71, 1.04) | 0.120 | -0.16 (-0.38, 0.05) | 0.134 |
| Obesity |  |  |  |  |  |  |
| If about average weight | 1.46 (1.32, 1.60) | <0.001 | -- |  | -- |  |
| If thinner | 1.48 (1.31, 1.67) | <0.001 | 1.01 (0.87, 1.18) | 0.847 | 0.03 (-0.16, 0.22) | 0.759 |
| If plumper | 1.48 (1.26, 1.73) | <0.001 | 1.01 (0.84, 1.22) | 0.885 | 0.08 (-0.16, 0.31) | 0.522 |
| LRT vs model without interactions (p=0.330) |  |  |  |  |  |  |

LRT, likelihood-ratio test; RERI, relative excess risk due to interaction

Referent: normal weight in adulthood.

Models adjusted for age, ethnicity, relative age voice break (males) or age at menarche (females), comparative height at age 10 years, alcohol status, smoking status, sleep pattern, diet pattern, self-rated health, employment status, and Townsend index quintile.

Supplementary Table 20. Associations of adulthood overweight and obesity with incident obesity-related cancer according to, and testing for effect modification by, comparative body weight at age 10 years (adjusted for adulthood variables including MET minutes per week for moderate and vigorous physical activity)

|  | Males | | | | | |
| --- | --- | --- | --- | --- | --- | --- |
|  | Stratum-specific estimate | | Interaction | | RERI | |
|  | HR (95% CI) | P-value | HR (95% CI) | P-value | Estimate (95% CI) | P-value |
| Overweight |  |  |  |  |  |  |
| If about average weight | 1.25 (1.11, 1.41) | <0.001 | -- |  | -- |  |
| If thinner | 1.23 (1.08, 1.41) | 0.002 | 0.98 (0.82, 1.18) | 0.866 | -0.02 (-0.22, 0.17) | 0.804 |
| If plumper | 1.14 (0.84, 1.55) | 0.409 | 0.91 (0.65, 1.27) | 0.576 | -0.10 (-0.48, 0.27) | 0.583 |
| Obesity |  |  |  |  |  |  |
| If about average weight | 1.57 (1.38, 1.80) | <0.001 | -- |  | -- |  |
| If thinner | 1.57 (1.34, 1.83) | <0.001 | 1.00 (0.82, 1.22) | 0.982 | -0.02 (-0.27, 0.24) | 0.907 |
| If plumper | 1.56 (1.15, 2.11) | 0.004 | 0.99 (0.71, 1.38) | 0.957 | 0.04 (-0.36, 0.43) | 0.861 |
| LRT vs model without interactions (p=0.933) |  |  |  |  |  |  |

|  | Females | | | | | |
| --- | --- | --- | --- | --- | --- | --- |
|  | Stratum-specific estimate | | Interaction | | RERI |  |
|  | HR (95% CI) | P-value | HR (95% CI) | P-value | Estimate (95% CI) | P-value |
| Overweight |  |  |  |  |  |  |
| If about average weight | 1.20 (1.08, 1.32) | <0.001 | -- |  | -- |  |
| If thinner | 1.09 (0.96, 1.23) | 0.183 | 0.91 (0.78, 1.07) | 0.248 | -0.11 (-0.28, 0.07) | 0.236 |
| If plumper | 1.00 (0.83, 1.22) | 0.968 | 0.84 (0.68, 1.04) | 0.117 | -0.19 (-0.44, 0.06) | 0.131 |
| Obesity |  |  |  |  |  |  |
| If about average weight | 1.49 (1.33, 1.66) | <0.001 | -- |  | -- |  |
| If thinner | 1.47 (1.28, 1.70) | <0.001 | 0.99 (0.83, 1.18) | 0.929 | 0.00 (-0.23, 0.24) | 0.988 |
| If plumper | 1.43 (1.19, 1.72) | <0.001 | 0.96 (0.78, 1.19) | 0.741 | 0.00 (-0.27, 0.28) | 0.982 |
| LRT vs model without interactions (p=0.440) |  |  |  |  |  |  |

LRT, likelihood-ratio test; RERI, relative excess risk due to interaction

Referent: normal weight in adulthood.

Models adjusted for age, ethnicity, relative age voice break (males) or age at menarche (females), comparative height at age 10 years, alcohol status, smoking status, sleep pattern, diet pattern, self-rated health, employment status, Townsend index quintile, and MET minutes per week for moderate and vigorous physical activity.

Supplementary Table 21. Models for incident obesity-related cancer examining the interaction between adult BMI and comparative body weight at age 10 years

|  | Males | | Females | |
| --- | --- | --- | --- | --- |
|  | HR (95% CI) | P-value | HR (95% CI) | P-value |
| Adult BMI | 1.05 (1.04, 1.06) | <0.001 | 1.04 (1.03, 1.05) | <0.001 |
| Comparative child body weight |  |  |  |  |
| About average weight (referent) | -- |  | -- |  |
| Thinner | 0.98 (0.92, 1.05) | 0.613 | 1.03 (0.97, 1.09) | 0.371 |
| Plumper | 1.04 (0.94, 1.15) | 0.480 | 1.02 (0.94, 1.10) | 0.683 |
| Interactions |  |  |  |  |
| Adult BMI*Thinner | 1.00 (0.98, 1.01) | 0.698 | 1.00 (0.99, 1.01) | 0.821 |
| Adult BMI*Plumper | 1.00 (0.98, 1.01) | 0.571 | 1.00 (0.99, 1.01) | 0.667 |

Models adjusted for age, ethnicity, relative age voice break (males) or age at menarche (females), and comparative height at age 10 years.

Supplementary Table 22. Associations of adulthood overweight and obesity with incident obesity-related cancer according to, and testing for effect modification by, comparative body weight at age 10 years (defined using the child polygenic risk score)

|  | Males | | | | | |
| --- | --- | --- | --- | --- | --- | --- |
|  | Stratum-specific estimate | | Interaction | | RERI | |
|  | HR (95% CI) | P-value | HR (95% CI) | P-value | Estimate (95% CI) | P-value |
| Overweight |  |  |  |  |  |  |
| If average (using child PRS) | 1.36 (1.22, 1.52) | <0.001 | -- |  | -- |  |
| If thinner (using child PRS) | 1.15 (1.01, 1.31) | 0.036 | 0.84 (0.71, 1.00) | 0.052 | -0.21 (-0.41, -0.02) | 0.033 |
| If plumper (using child PRS) | 1.32 (1.06, 1.65) | 0.012 | 0.97 (0.76, 1.24) | 0.812 | -0.02 (-0.30, 0.26) | 0.897 |
| Obesity |  |  |  |  |  |  |
| If average (using child PRS) | 1.76 (1.56, 1.97) | <0.001 | -- |  | -- |  |
| If thinner (using child PRS) | 1.70 (1.48, 1.96) | <0.001 | 0.97 (0.81, 1.16) | 0.740 | -0.05 (-0.29, 0.20) | 0.702 |
| If plumper (using child PRS) | 1.63 (1.30, 2.05) | <0.001 | 0.93 (0.72, 1.20) | 0.582 | -0.08 (-0.41, 0.26) | 0.661 |
| LRT vs model without interactions (p=0.159) |  |  |  |  |  |  |

|  | Females | | | | | |
| --- | --- | --- | --- | --- | --- | --- |
|  | Stratum-specific estimate | | Interaction | | RERI |  |
|  | HR (95% CI) | P-value | HR (95% CI) | P-value | Estimate (95% CI) | P-value |
| Overweight |  |  |  |  |  |  |
| If average (using child PRS) | 1.06 (0.97, 1.16) | 0.163 | -- |  | -- |  |
| If thinner (using child PRS) | 1.26 (1.13, 1.40) | <0.001 | 1.18 (1.03, 1.36) | 0.019 | 0.18 (0.03, 0.32) | 0.015 |
| If plumper (using child PRS) | 1.17 (1.00, 1.36) | 0.053 | 1.10 (0.92, 1.31) | 0.317 | 0.09 (-0.09, 0.26) | 0.334 |
| Obesity |  |  |  |  |  |  |
| If average (using child PRS) | 1.42 (1.30, 1.56) | <0.001 | -- |  | -- |  |
| If thinner (using child PRS) | 1.59 (1.41, 1.79) | <0.001 | 1.12 (0.96, 1.30) | 0.150 | 0.12 (-0.06, 0.31) | 0.193 |
| If plumper (using child PRS) | 1.86 (1.59, 2.16) | <0.001 | 1.30 (1.09, 1.56) | 0.004 | 0.35 (0.13, 0.57) | 0.002 |
| LRT vs model without interactions (p=0.007) |  |  |  |  |  |  |

LRT, likelihood-ratio test; PRS, polygenic risk score; RERI, relative excess risk due to interaction

Referent: normal weight in adulthood.

Models adjusted for age, ethnicity, relative age voice break (males) or age at menarche (females), and comparative height at age 10 years.

Supplementary Table 23. Models for incident obesity-related cancer examining 1) the interaction between child and adult polygenic risk scores and 2) interactions between adulthood weight status and the child polygenic risk score

|  | Males | | Females | |
| --- | --- | --- | --- | --- |
|  | HR (95% CI) | P-value | HR (95% CI) | P-value |
| Child PRS (mean 0, SD 1) | 1.04 (1.01, 1.07) | 0.016 | 1.00 (0.97, 1.03) | 0.947 |
| Adult PRS (mean 0, SD 1) | 1.04 (1.01, 1.07) | 0.008 | 1.01 (0.98, 1.04) | 0.498 |
| Interaction |  |  |  |  |
| Child PRS*Adult PRS | 0.99 (0.96, 1.01) | 0.326 | 1.02 (0.99, 1.04) | 0.154 |
| Adult weight status |  |  |  |  |
| Normal weight (referent) | -- |  | -- |  |
| Overweight | 1.28 (1.18, 1.39) | <0.001 | 1.14 (1.07, 1.22) | <0.001 |
| Obesity | 1.72 (1.58, 1.87) | <0.001 | 1.55 (1.45, 1.65) | <0.001 |
| Child PRS (mean 0, SD 1) | 1.03 (0.96, 1.10) | 0.422 | 0.99 (0.95, 1.04) | 0.816 |
| Interactions |  |  |  |  |
| Overweight*Child PRS | 1.04 (0.96, 1.12) | 0.361 | 0.97 (0.91, 1.03) | 0.274 |
| Obesity*Child PRS | 0.98 (0.90, 1.06) | 0.614 | 1.04 (0.97, 1.11) | 0.281 |

PRS, polygenic risk score

Models adjusted for age, ethnicity, relative age voice break (males) or age at menarche (females), and comparative height at age 10 years.

Supplementary Table 24. Associations of adulthood overweight and obesity with incident obesity-related cancer according to, and testing for effect modification by, comparative body weight at age 10 years: observational and genetic analyses in participants with White ethnicity

|  | Males | | | | | |
| --- | --- | --- | --- | --- | --- | --- |
|  | Stratum-specific estimate | | Interaction | | RERI | |
|  | HR (95% CI) | P-value | HR (95% CI) | P-value | Estimate (95% CI) | P-value |
| Overweight |  |  |  |  |  |  |
| If average | 1.25 (1.12, 1.40) | <0.001 | -- |  | -- |  |
| If thinner | 1.27 (1.12, 1.43) | <0.001 | 1.01 (0.86, 1.20) | 0.886 | 0.01 (-0.17, 0.18) | 0.933 |
| If plumper | 1.27 (0.95, 1.71) | 0.111 | 1.01 (0.74, 1.39) | 0.928 | 0.02 (-0.32, 0.37) | 0.888 |
| Obesity |  |  |  |  |  |  |
| If average | 1.67 (1.48, 1.89) | <0.001 | -- |  | -- |  |
| If thinner | 1.68 (1.46, 1.93) | <0.001 | 1.00 (0.83, 1.21) | 0.970 | -0.01 (-0.26, 0.23) | 0.904 |
| If plumper | 1.72 (1.29, 2.29) | <0.001 | 1.03 (0.75, 1.40) | 0.874 | 0.07 (-0.30, 0.43) | 0.720 |
| LRT vs model without interactions (p>0.999) |  |  |  |  |  |  |
| Overweight (using adult PRS) |  |  |  |  |  |  |
| If average (using child PRS) | 1.12 (1.01, 1.25) | 0.035 | -- |  | -- |  |
| If thinner (using child PRS) | 1.08 (0.97, 1.21) | 0.156 | 0.96 (0.82, 1.13) | 0.646 | -0.04 (-0.20, 0.12) | 0.616 |
| If plumper (using child PRS) | 0.97 (0.75, 1.25) | 0.827 | 0.86 (0.66, 1.14) | 0.303 | -0.16 (-0.49, 0.17) | 0.351 |
| Obesity (using adult PRS) |  |  |  |  |  |  |
| If average (using child PRS) | 1.27 (1.13, 1.43) | <0.001 | -- |  | -- |  |
| If thinner (using child PRS) | 0.97 (0.83, 1.14) | 0.730 | 0.76 (0.63, 0.93) | 0.008 | -0.30 (-0.52, -0.08) | 0.007 |
| If plumper (using child PRS) | 0.97 (0.75, 1.25) | 0.800 | 0.76 (0.57, 1.01) | 0.057 | -0.31 (-0.65, 0.03) | 0.077 |
| LRT vs model without interactions (p=0.035) |  |  |  |  |  |  |

|  | Females | | | | | |
| --- | --- | --- | --- | --- | --- | --- |
|  | Stratum-specific estimate | | Interaction | | RERI |  |
|  | HR (95% CI) | P-value | HR (95% CI) | P-value | Estimate (95% CI) | P-value |
| Overweight |  |  |  |  |  |  |
| If average | 1.17 (1.08, 1.28) | <0.001 | -- |  | -- |  |
| If thinner | 1.16 (1.04, 1.29) | 0.007 | 0.99 (0.86, 1.14) | 0.879 | -0.01 (-0.16, 0.14) | 0.903 |
| If plumper | 0.98 (0.82, 1.16) | 0.785 | 0.83 (0.69, 1.01) | 0.060 | -0.20 (-0.42, 0.02) | 0.071 |
| Obesity |  |  |  |  |  |  |
| If average | 1.53 (1.39, 1.68) | <0.001 | -- |  | -- |  |
| If thinner | 1.57 (1.39, 1.77) | <0.001 | 1.03 (0.88, 1.20) | 0.723 | 0.06 (-0.15, 0.27) | 0.572 |
| If plumper | 1.52 (1.30, 1.78) | <0.001 | 0.99 (0.83, 1.20) | 0.952 | 0.06 (-0.18, 0.31) | 0.602 |
| LRT vs model without interactions (p=0.211) |  |  |  |  |  |  |
| Overweight (using adult PRS) |  |  |  |  |  |  |
| If average (using child PRS) | 0.96 (0.88, 1.04) | 0.306 | -- |  | -- |  |
| If thinner (using child PRS) | 1.06 (0.95, 1.17) | 0.307 | 1.10 (0.97, 1.26) | 0.150 | 0.10 (-0.03, 0.23) | 0.145 |
| If plumper (using child PRS) | 1.08 (0.91, 1.28) | 0.355 | 1.13 (0.94, 1.37) | 0.199 | 0.12 (-0.06, 0.30) | 0.189 |
| Obesity (using adult PRS) |  |  |  |  |  |  |
| If average (using child PRS) | 1.01 (0.92, 1.11) | 0.834 | -- |  | -- |  |
| If thinner (using child PRS) | 0.92 (0.79, 1.07) | 0.281 | 0.91 (0.76, 1.09) | 0.307 | -0.09 (-0.26, 0.08) | 0.305 |
| If plumper (using child PRS) | 1.18 (1.00, 1.40) | 0.051 | 1.17 (0.96, 1.42) | 0.113 | 0.16 (-0.03, 0.35) | 0.103 |
| LRT vs model without interactions (p=0.090) |  |  |  |  |  |  |

LRT, likelihood-ratio test; PRS, polygenic risk score; RERI, relative excess risk due to interaction

Referent: normal weight in adulthood.

Models adjusted for age, ethnicity, relative age voice break (males) or age at menarche (females), and comparative height at age 10 years.
